# Lipid droplet-associated gene signatures classify metabolic subtypes and identify PLIN3 as a key driver in hepatocellular carcinoma

**DOI:** 10.1016/j.gendis.2026.102067

**Published:** 2026-02-03

**Authors:** Huiying Gu, Qiumin Wu, Haibei Zhao, JingLong Du, Zhenzhen Zhang, Juan Chen

**Affiliations:** a Department of Infectious Disease, Children’s Hospital of Chongqing Medical University, National Clinical Research Center for Children and Adolescents’ Health and Diseases, Ministry of Education Key Laboratory of Child Development and Disorders, Chongqing Key Laboratory of Child Rare Diseases in Infection and Immunity, Chongqing 400014, China; b The Key Laboratory of Molecular Biology of Infectious Diseases Designated by the Chinese Ministry of Education, Chongqing Medical University, Chongqing 400016, China; c College of Medical Informatics, Chongqing Medical University, Chongqing 400016, China; d College of Laboratory Medicine, Chongqing Medical University, Chongqing 400016, China

**Keywords:** Hepatocellular carcinoma, LDAG, Lipid droplet, Molecular subtype, PLIN3

## Abstract

Hepatocellular carcinoma is characterized by considerable molecular heterogeneity, which complicates prognostic predictions and contributes to therapeutic resistance. This study aimed to develop a molecular classification framework grounded in lipid droplet-associated genes (LDAGs) and to comprehensively elucidate their biological significance and clinical applicability in guiding personalized treatment approaches. By leveraging multi-cohort datasets, we defined LDAG-based molecular subtypes and systematically characterized their genomic alterations, metabolic features, pathway activation patterns, and therapeutic vulnerabilities. Three distinct subtypes (C1–C3) were identified according to LDAG expression patterns, each demonstrating unique clinical outcomes, mutational profiles, and metabolic reprogramming. The C1 subtype correlated with the poorest overall survival, more advanced tumor stages, and activation of pro-proliferative signaling pathways. Therapeutic vulnerabilities were subtype-dependent, with C1 showing heightened sensitivity to sorafenib. Five pivotal LDAGs (PLIN3, SET, CKAP4, RAP1B, and PISD) were implicated in the aggressive phenotype of C1, among which PLIN3 exhibited the strongest prognostic value. Functional assays confirmed that PLIN3 knockdown reduced lipid accumulation, suppressed cell proliferation and migration, and impaired tumorigenesis, whereas its overexpression promoted aggressive tumor behavior. In conclusion, our LDAG-based classification system stratifies hepatocellular carcinoma into three clinically relevant subtypes. PLIN3 emerges as a promising prognostic biomarker and therapeutic target, thereby mechanistically linking lipid metabolism to hepatocellular carcinoma progression.

## Introduction

Hepatocellular carcinoma (HCC) is a major global health concern, responsible for 90% of primary liver cancers and the third-highest cancer-related mortality rate worldwide.^1^ While molecularly targeted therapies, such as multikinase inhibitors (*e.g.*, sorafenib and lenvatinib) and immune checkpoint inhibitors, have expanded treatment options for advanced HCC, their clinical efficacy remains limited by pervasive therapeutic resistance and tumor heterogeneity 2, 3, 4. Tumor heterogeneity operates across multiple biological strata, such as recurrent genomic alterations in driver genes (*e.g.*, TP53 inactivation, CTNNB1 mutation, TERT promoter amplification), dynamic epigenetic remodeling of chromatin states, and spatially organized immune-microenvironmental niches that collectively enable therapeutic evasion 5, 6, 7. This complexity critically undermines the predictive utility of conventional histopathological classifications.

Advances in multiomics profiling are revolutionizing the molecular taxonomy of HCC. For example, Fan et al conducted a proteomic analysis of HBV-related HCC, identifying three distinct subgroups with unique characteristics in metabolic reprogramming, microenvironment dysregulation, cell proliferation, and potential therapeutic targets.^8^ Subsequent single-cell transcriptomic studies further resolved HCC ecosystems into three transcriptionally unique cellular subtypes, including the metab-subtype, prol-subtype, and EMT-subtype.^9^ These findings underscore the essential role of omics-based molecular stratification in informing therapeutic strategies.

Emerging insights into cancer metabolism have redefined lipid droplets (LDs) as dynamic metabolic hubs orchestrating bioenergetic plasticity, redox homeostasis, and oncogenic signaling 10, 11, 12, 13. Mechanistically, tumor cells exploit LD accumulation through three synergistic pathways: ATP generation via compartmentalized β-oxidation during nutrient stress, membrane biosynthesis through spatially regulated phospholipid production, and detoxification of cytotoxic lipid peroxides via polyunsaturated fatty acid sequestration.^10^^,^^13^ This metabolic adaptation is governed by an evolutionarily conserved LD-associated proteome, including perilipins (PLIN1-5), lipases (ATGL/HSL), and hypoxia-responsive regulators (HILPDA), whose dysregulation creates therapeutic vulnerabilities 13, 14, 15, 16, 17. In HCC, transcriptomic analyses have revealed ≥ 2-fold dysregulation of LD-associated genes (LDAGs) in 63% of tumors compared with nontumor tissue, suggesting that aberrant LDAG expression may represent a hallmark of hepatocarcinogenesis.^18^ However, the lack of a unified framework integrating LD-centric subtypes with established HCC classifications continues to hinder clinical translation.

In this study, we comprehensively profiled LDAG expression patterns across 1834 HCC samples, identifying three molecular subtypes with divergent clinical outcomes, mutational landscapes, and metabolic features. The aggressive C1 subtype exhibited advanced disease stages, vascular invasion, and significantly reduced median overall survival. We further propose subtype-specific therapeutic strategies for the aggressive C1 subtype. Functional characterization identified five hub LDAGs (PLIN3, SET, CKAP4, RAP1B, and PISD) as central regulators of C1 pathogenesis. Notably, PLIN3 has emerged as a master coordinator of HCC progression through coordinated modulation of lipid metabolism and oncogenic signaling. Our study establishes an LDAG-driven classification system that synergizes with TCGA subtypes, offering prognostic stratification and actionable targets for precision therapy.

## Materials and methods

### Collection of LDAGs

Human LDAGs were identified via the Gene Ontology (GO) database via the AmiGO 2 platform (http://amigo.geneontology.org/amigo). A search was conducted using the keyword “lipid droplet” under the species “*Homo sapiens*”, which yielded six related GO terms: “lipid droplet”, “lipid droplet disassembly”, “protein localization to lipid droplet”, “lipid droplet organization”, “lipid droplet fusion”, and “lipid droplet formation”. The proteins associated with these six terms were compiled into a unified set, resulting in 122 LD-associated proteins. The comprehensive list of LDAGs utilized in this study is available in Table S1.

### Public data collection and preparation

This study analyzed 12 publicly available datasets, including nine microarray datasets and three RNA-sequencing (RNA-seq) datasets. A summary of these datasets is provided in Table S3. HCC patient data were acquired from prominent repositories, including The Cancer Genome Atlas (TCGA), the Gene Expression Omnibus (GEO), and the International Cancer Genome Consortium (ICGC). All datasets comprised both clinical and sequencing data, sourced from the UCSC Xena Browser, GEO database, ICGC Data Portal, and ArrayExpress. Somatic mutation data (MAF files) for the TCGA-LIHC cohort were also retrieved from the TCGA repository. RNA sequencing data in STAR-counts format, along with clinical information, were processed and converted into transcripts per million (TPM) format. These data underwent log transformation for normalization using the log_2_(TPM+1) method.

The analysis of LDAGs utilized nine patient cohorts, including TCGA-LIHC, LIRI-JP, and several GEO cohorts: GSE36376, GSE25097, GSE45436, GSE102083, GSE112790, GSE124535, GSE14520, GSE116174, GSE36133, and E-MTAB-3610. The study included 1834 HCC samples, consisting of 371 from the TCGA-LIHC cohort, 243 from the ICGC-LIRI-JP cohort, and additional samples from GEO cohorts.

We concentrated on the TCGA-LIHC, ICGC-LIRI-JP, GSE14520, and GSE116174 datasets for survival analysis and external validation. Additionally, the GSE36133 and E-MTAB-3610 datasets were utilized to explore potential subgroup-specific drug targets. These datasets provided a robust foundation for investigating the role of LDAGs in HCC heterogeneity and their potential as prognostic biomarkers and therapeutic targets.

### Differential expression analysis

Differential gene expression analysis was conducted using the limma package in R (version 3.40.6), which applies linear models for microarray data (doi: 10.1093/nar/gkv007).^19^ The lmFit function was employed for multiple linear regression, and the eBayes function was utilized for empirical Bayes moderation to identify differentially expressed genes (DEGs) between experimental and control groups.

### Consensus molecular clustering

HCC subclass identification utilized three cohorts with available clinical outcomes: TCGA-LIHC, ICGC-LIRI-JP, and GSE14520. The TCGA-LIHC and ICGC datasets utilized RNA sequencing, whereas the GSE14520 cohort’s transcriptome data were obtained from microarray analysis. HCC samples from the cohorts were categorized using consensus clustering based on the expression of 122 LDAGs.^20^ The ConsensusClusterPlus R package was employed to identify the optimal number of clusters (k), with parameters configured as reps = 1000, pItem = 0.8, and pFeature = 1. The Bioconductor package complexHeatmap generated heatmaps.^21^ Based on the assessment of clustering stability through the cumulative distribution function (CDF) and delta area plots, three clusters were selected. These clusters were categorized according to LDAG levels: Cluster 1 (C1) represented high levels, Cluster 2 (C2) represented intermediate levels, and Cluster 3 (C3) represented low levels (Table S5). Principal component analysis was employed to assess the quality of clustering. The principal component analysis was performed using the stats package in R (version 3.6.0). Specifically, expression profiles were standardized using z-scores, and the prcomp function was utilized to conduct dimension reduction analysis, yielding the reduced-dimension matrix.

### Functional enrichment analysis

Functional enrichment analyses, including Kyoto Encyclopedia of Genes and Genomes (KEGG) and GO, were performed using the R package ggplot2 (version 3.5.0).^22^ Gene set function enrichment analysis was conducted utilizing the KEGG REST API to obtain updated KEGG pathway gene annotations, and the R package org. Hs. eg, db (version 3.1.0) was employed for GO gene annotations as the background. The R package clusterProfiler (version 3.14.3) facilitated the mapping of genes to the background set and the execution of enrichment analysis, resulting in gene set enrichment outcomes. Parameters were set with a minimum gene set size of 5 and a maximum of 5000, and *P*-values < 0.05 were deemed statistically significant.

### Survival analysis

Survival analyses were conducted to categorize patients into subgroups using Consensus Clustering or by applying median values as thresholds for continuous variables. The Kaplan–Meier method was employed for survival analysis, and the log-rank test was used to evaluate the statistical significance of differences. Hazard ratios were estimated using Cox regression models from the survival R package. Both univariate and multivariate survival analyses were performed using Cox proportional hazards regression. In the multivariate Cox regression analysis, only variables with a *P*-value less than 0.05 from the univariate analysis were included. Survival curves and time-dependent ROC curves were generated utilizing the survminer and timeROC R packages, respectively.

### Molecular features of HCC subtypes

To identify the DEGs among the three clusters, we conducted pairwise comparisons of each cluster against the other two clusters. DEGs were identified using the limma package with criteria of *P* < 0.05 and |log_2_FC| > 0.585. DEGs commonly expressed across all three clusters were visualized using Venn diagrams. The DEGs set was then used to perform the second cluster analysis.

### Exploration of the molecular differences among the three LDAG-associated HCC subtypes

To identify cluster-specific DEGs, we conducted further screening of DEGs, defining those with a *P*-value less than 0.05 and an absolute log_2_ (fold change) greater than 1 as cluster-specific DEGs. The gene set variation analysis (GSVA) R package (version 1.40.1) was employed to compute enrichment scores for each sample within the gene sets. Gene expression profiles were used to establish predefined gene rankings, following the methodology described by Hänzelmann et al.^23^ Predefined gene sets, comprising between 5 and 5000 genes, were utilized to assess enrichment scores. An enrichment score matrix was generated by calculating the enrichment score for each sample across all gene sets. GSVA was applied to calculate activity scores for KEGG and 50 hallmark gene sets, with the latter obtained from the MsigDB database (http://www.gsea-msigdb.org/gsea/msigdb/index.jsp). Distinct pathways among the three HCC clusters were visualized using the ComplexHeatmap R package.

### Mutational profile analysis

Somatic mutation data from the TCGA dataset (mc3.v0.2.8.PUBLIC.maf.gz) were downloaded from Xena. The MAF file was summarized and analyzed using the R package maftools.^24^ The ComplexHeatmap package’s oncoPrint function was used for visualization.

### Correlation analysis

To highlight the significance of LDAGs (LD-DEGs) and non-LD-DEGs in Cluster 1 within the gene network of HCC progression, highly relevant regulated genes in Cluster 1 (correlation coefficient >0.5 and *P*-value < 0.05) were identified using the “tinyarray” package in R.

### Estimating drug sensitivity in clinical cohorts

Drug responses in clinical samples were evaluated utilizing three pharmacogenomic datasets of human cancer cell lines: PRISM (https://depmap.org/portal/prism/), the Cancer Therapeutics Response Portal (CTRP; https://portals.broadinstitute.org/ctrp), and Genomics of Drug Sensitivity in Cancer (GDSC; https://www.cancerrxgene.org/). Drug sensitivity was quantified using the area under the dose–response curve (AUC), where lower AUC values indicate increased sensitivity. The curated datasets were acquired from the Functional Data Consistency Explorer (FDCE) web tool.^25^ Drugs with missing data in over 20% of cell lines and cell lines with missing data for more than 50% of drugs were excluded from analysis. The PRISM dataset includes 479 cancer cell lines and 1434 drugs, while the CTRP dataset comprises 845 cancer cell lines and 405 drugs, and the GDSC dataset contains 707 cancer cell lines and 285 drugs.

The overlaps of drugs and cell lines across the three datasets were illustrated in Figure 3. Expression data for cancer cell lines were obtained from the Cancer Cell Line Encyclopedia (CCLE) for the CTRP and PRISM datasets, and from ArrayExpress (E-MTAB-3610) for the GDSC dataset.^26^ The ridge regression model, as implemented in the oncoPredict R package, was employed to estimate drug responses in HCC patients.^27^ Drugs were classified as exhibiting a differential response if they demonstrated a *P*-value of less than 0.05 across all comparisons: C1 *vs*. C2+C3, C2 *vs*. C1+C3, and C3 *vs*. C1+C2.

**Figure 1:**
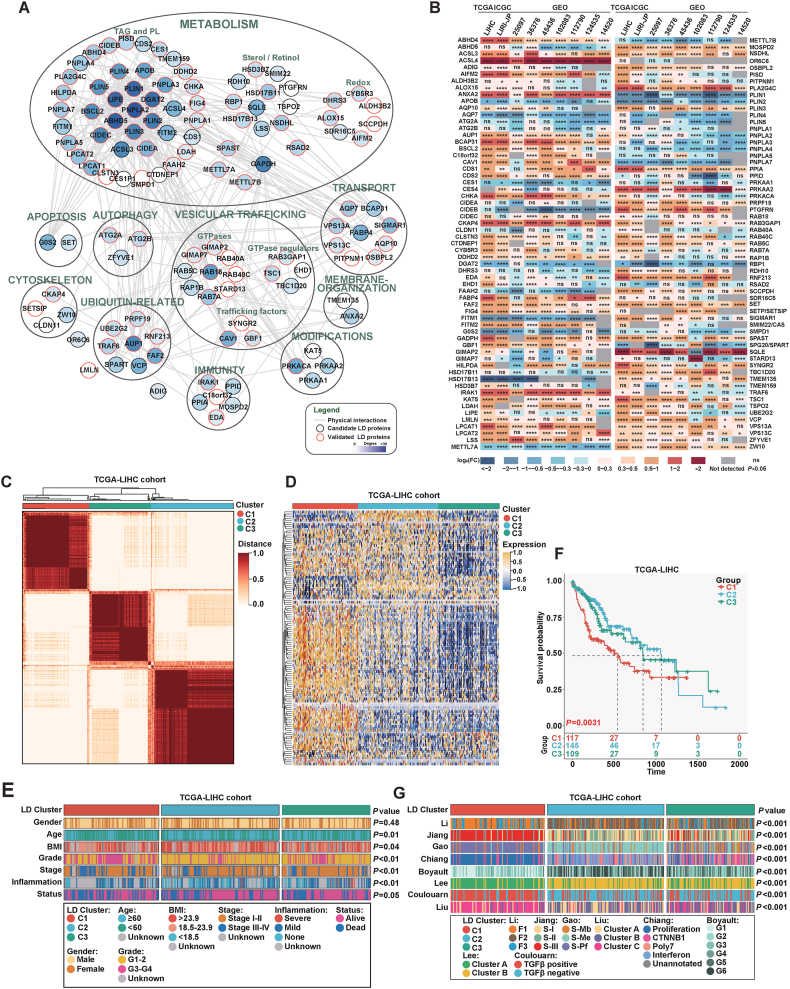
Characterization of lipid droplet-associated genes (LDAGs) in hepatocellular carcinoma (HCC) and identification of LDAG-associated HCC subclasses. **(A)** Clustering of LD-associated proteins based on their biological roles. Proteins are grouped into functional modules based on Gene Ontology (GO) analysis and UniProt functional annotations. The solid lines represent physical interactions, as annotated in the BioGRID database. The intensity of the blue color in each node corresponds to the confidence score for the interaction. The nodes outlined in red represent proteins for which LD localization has been experimentally validated, whereas the nodes outlined in black represent proteins for which LD localization has not been validated. **(B)** Heatmap displaying the expression profiles of the 122 LDAGs across several HCC datasets. The color intensity represents the differential expression of each gene in the HCC and control samples within the dataset. **(C)** Consensus matrix heatmap based on the gene expression data of 122 LDAGs in the TCGA-LIHC cohort. The color coding represents the three distinct HCC subclasses: Cluster 1 (C1), Cluster 2 (C2), and Cluster 3 (C3). **(D)** Heatmap showing the expression patterns of LDAGs across the three identified HCC subgroups. **(E)** Clinical characteristics of the LDAG-associated HCC subgroups in the TCGA-LIHC cohort. **(F)** Kaplan–Meier survival analysis of the three subgroups in the TCGA-LIHC dataset. **(G)** Comparisons of the LDAG subtypes with eight previously reported classifications based on the TCGA data set. Statistical significance is indicated as “ns” (no significant difference); ∗*P* < 0.05, ∗∗*P* < 0.01, ∗∗∗*P* < 0.001, and ∗∗∗∗*P* < 0.0001.

**Figure 2:**
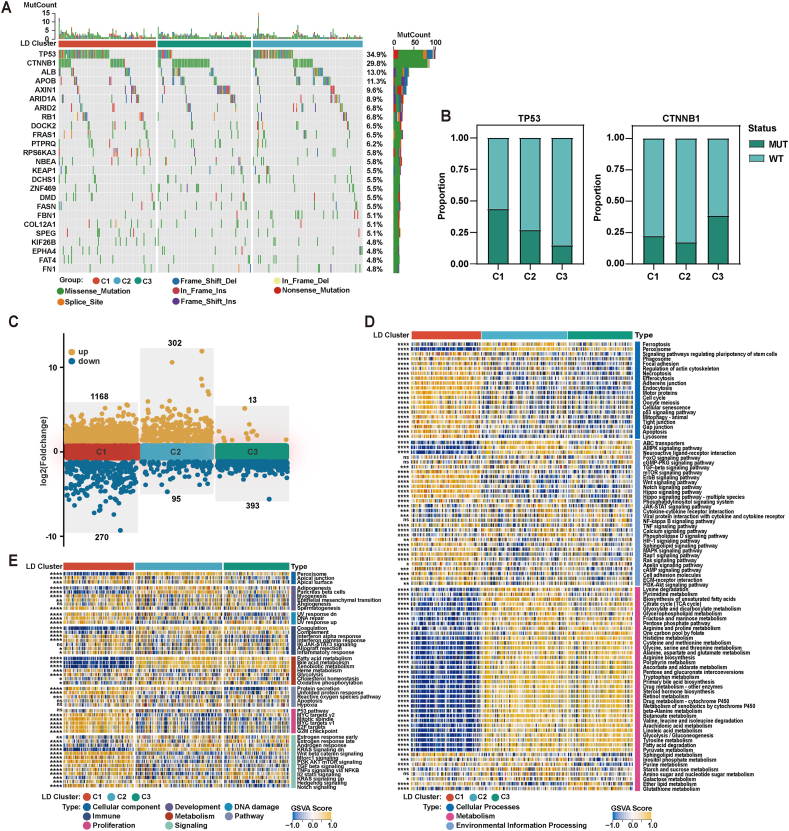
Genomic and signaling landscape in the lipid droplet-associated gene (LDAG) subtypes. **(A)** Oncoplot depicting the top 20 somatic mutations of known hepatocellular carcinoma (HCC) driver genes. **(B)** The percentage of TP53 and CTNNB1 mutations within LDAG-associated HCC subgroups. **(C)** Bar plot illustrating the cluster-specific differentially expressed genes (DEGs) identified for each of the three HCC subtypes in the TCGA-LIHC cohort. The red dots represent upregulated genes, and the blue dots represent downregulated genes. **(D)** Heatmap displaying the gene set variation analysis (GSVA) scores of the Kyoto Encyclopedia of Genes and Genomes (KEGG) pathway enrichment analysis for DEGs across the three clusters. **(E)** Heatmap displaying the GSVA scores for 50 hallmark gene sets across the three clusters. C1, Cluster 1; C2, Cluster 2; and C3, Cluster 3. Statistical significance is indicated as “ns” (no significant difference); ∗*P* < 0.05, ∗∗*P* < 0.01, ∗∗∗*P* < 0.001, and ∗∗∗∗*P* < 0.0001.

**Figure 3:**
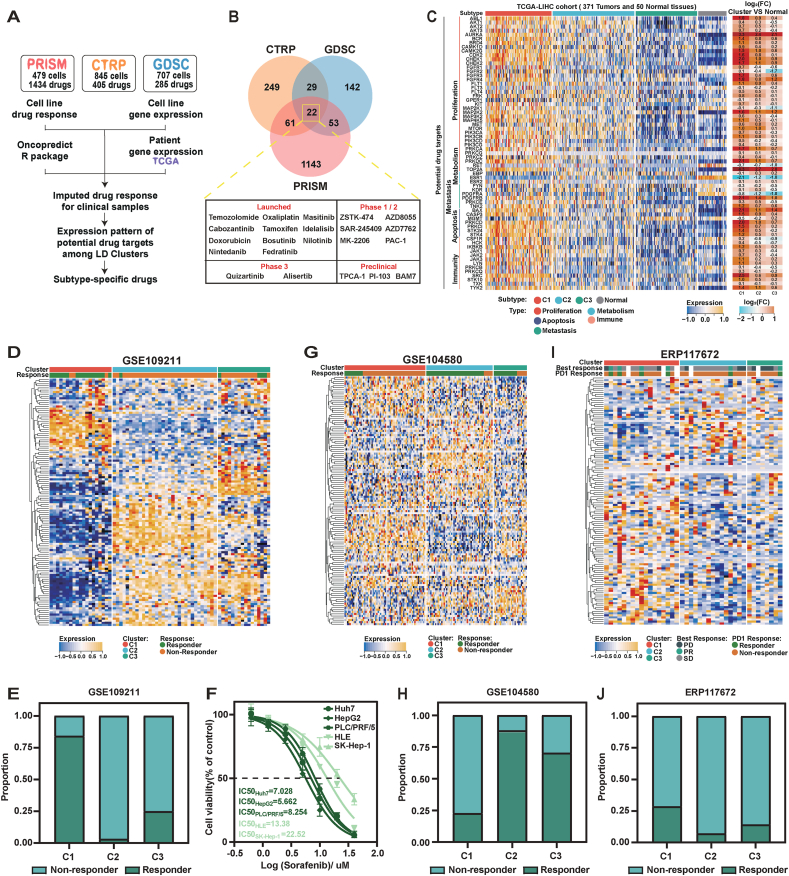
Therapeutic vulnerabilities in the lipid droplet-associated gene (LDAG) subtypes. **(A)** Overview of the analysis procedures used to identify subtype-specific drug targets for hepatocellular carcinoma. **(B)** Venn diagram summarizing the C1-specific drugs identified in the analysis. **(C)** Expression patterns of potential drug targets in different subtypes and nontumor tissues from the TCGA-LIHC dataset. The right panel shows the log_2_ (fold change) in expression for different subtypes versus nontumor tissues. Drug target information was curated from the Drug Repurposing Hub. **(D)** Heatmap showing the 3 LDAG subtypes in the GSE109211 data set with sorafenib response available. **(E)** The percentage of sorafenib responders and non-responders across LDAG subtypes in the indicated data set. **(F)** Dose‒response analyses of sorafenib sensitivity in liver cancer cell lines following a 2-day treatment period. The cell lines were classified as relatively sensitive (IC50 < 10 μM) or resistant (IC50 > 10 μM). **(G)** Heatmap showing the 3 LDAG subtypes in the GSE104580 dataset. **(H)** The percentage of trans-arterial chemoembolization (TACE) responders and non-responders across LDAG subtypes in GSE104580. **(I)** Heatmap showing the 3 LDAG subtypes in the ERP117672 dataset. **(J)** The percentage of PD1 responders and non-responders across LDAG subtypes in ERP117672. C1, Cluster 1; C2, Cluster 2; and C3, Cluster 3.

### Drug target analysis

Information on 6798 compounds was sourced from the Drug Repurposing Hub (https://clue.io/repurposing, version 3/24/2020), which is a curated collection of FDA-approved drugs, clinical trial drugs, and pre-clinical tool compounds.^28^ After removing duplicates, 2183 drug targets were identified. We conducted differential expression analysis comparing each subtype with non-tumor tissue to pinpoint drug targets specific to each subtype.

### Antibodies and reagents

The antibodies utilized in this study included PLIN3 (10694-1-AP, Proteintech), PLIN2 (80362-2-RR, Proteintech), LAMP1 (A5106, Selleck), VDAC1 (ET1601-20, HUABIO), GAPDH (TA309157, ZSGB-BIO), β-Actin (81115-1-RR, Proteintech), HA-tag (201113, Zen-bio), F-actin (DyLight™ 488 Phalloidin, CST, 12935S), goat anti-rabbit IgG-HRP (NA934v, GE Healthcare), goat anti-mouse IgG-HRP (NA931v, GE Healthcare), goat anti-Rabbit IgG (H + L) cross-adsorbed secondary antibody, and Alexa Fluor™ 555 (A-21428, Thermo).

Other reagents used in this study included triglyceride quantification kit (BC0625, Solarbio), BODIPY™ 493/503 (D3922, Thermo), TRIzol (DP405, TIANGEN), T4 DNA Ligase (M0202, BioLabs), DAPI (D1306, Thermo), Mitomycin C (S8146, Selleck), SYBR Green (Bio-Rad), BeyoECL Plus (P0018S, Beyotime), and Lipofectamine™ 3000 (L3000001, Invitrogen).

### Clinical sample acquisition

Human HCC tissues and corresponding non-tumor liver tissues were acquired from the First Affiliated Hospital of Chongqing Medical University, China. Following resection, tissues were immediately snap-frozen in liquid nitrogen and stored at −80 °C for subsequent analyses. Tissues were homogenized using whole-cell lysis buffer for Western blotting analysis, while specimens for immunohistochemistry were formalin-fixed and paraffin-embedded. The study complied with the Declaration of Helsinki, and all participants provided written informed consent. The Ethics Committee of Chongqing Medical University approved the study protocol (approval number 2024028).

### Quantitative real-time PCR

Quantitative PCR was conducted using SYBR green dye on the QuantStudio 5 Real-Time PCR System (Applied Biosystems, Waltham, MA, USA). Total RNA was extracted with TRIzol and then reverse-transcribed to cDNA. Quantitative PCR was performed with primers for PLIN3 and β-actin, following a cycling protocol of 95 °C for 3 min, 40 cycles of 95 °C for 20 s, 60 °C for 20 s, and 72 °C for 20 s, with a final extension at 72 °C for 5 min. Data were analyzed using the 2^–ΔΔCt^ method with ACTIN as the internal control. All experiments were done in duplicate. The primers used were as follows: β-actin: Forward: 5′-CTCTTCCAGCCTTCCTTCCT-3′, Reverse: 5′-AGCACTGTGT TGGCGTACAG-3′. PLIN3: Forward: 5′-TATGCCTCCACCAAGGAGAG-3′, Reverse: 5′-ATTCGCTGGCTGATGCAATCT-3′. PISD: Forward: 5′-GTGAG GTGGAGCAGGTAA-3′, Reverse: 5′-GGAGTGGAAGCAGTGGTA-3′. CKAP4: Forward: 5′-AGAAGAGGAGCCAGAAGG-3′, Reverse: 5′-TCGGTGTAGATG TCAGACT-3′. RAP1B: Forward: 5′-TTCCATCACAGCACAGTC-3′, Reverse: 5′-TTCATCTTCCAAGTCACACT-3′. SET: Forward: 5′-CAACCATCCACAAGT GTCT-3′, Reverse: 5′-TTCTCCTTCTCCTTCTTCATC-3′.

### Western blotting analysis

Cells or tissues were lysed using RIPA buffer supplemented with a protease inhibitor cocktail (04693132001, Roche) to extract total protein. Protein concentrations were quantified utilizing the BCA assay kit (23227, Thermo, USA). Proteins were then equally loaded and resolved by SDS-PAGE, subsequently transferred to a PVDF membrane (Merck Millipore, USA), and blocked with 5% non-fat milk in TBST at room temperature for 2 h. The membranes were incubated with specific primary antibodies at 4 °C overnight. Following washing, membranes were incubated with species-specific horseradish peroxidase (HRP)-conjugated secondary antibodies, and protein blots were visualized using enhanced chemiluminescence (ECL) reagent (WBKLS0500, Millipore, USA). Densitometric analysis was conducted using ImageJ software.

### Immunohistochemistry

Immunohistochemistry was performed on 4-μm-thick paraffin-embedded sections. Sections were deparaffinized and rehydrated through a gradient ethanol series, and antigen retrieval was conducted using sodium citrate buffer. Endogenous peroxidase activity was inhibited using 3% H_2_O_2_ for 10 min following a 15-min treatment with 0.5% Triton X-100. Sections were treated with 10% normal goat serum for 1 h, followed by overnight incubation with specific primary antibodies at 4 °C. Following incubation, the tissue sections were treated with HRP-conjugated secondary antibodies, and immunoreactivity was visualized using diaminobenzidine staining. Nuclear counterstaining was conducted with hematoxylin. Immunohistochemical scores were calculated by multiplying the staining intensity (scored as 0 for negative, 1 for weak, 2 for moderate, and 3 for strong) by the percentage of cells exhibiting positive staining (scored as 0 for 0%, 1 for ≤ 10%, 2 for 10%–50%, and 3 for ≥ 50%).

### Small interfering RNA (siRNA) and expression plasmid construction

Two siRNAs targeting the five hub LDAGs (PLIN3, SET, CKAP4, RAP1B, and PISD) were designed using BLOCK-iT™ RNAi Designer and synthesized by Tsingke Biotechnology in Beijing, China. The sequences of the siRNAs were as follows: siPLIN3-1, sense 5′-GGAACAGAGCUACUUCGUA(dT) (dT)-3′, antisense 5′-UACGAAGUAGCUCUGUUCC(dT)(dT)-3′; siPLIN3-2, sense 5′-GGACCUGUCCAGCAGCAUU(dT)(dT)-3′, antisense 5′-AAUGCUGCUGG ACAGGUCC(dT)(dT)-3′. siSET-1, sense 5′-GACCAGAGUUGAAGUGACA (dT)(dT)-3′, antisense 5′-UGUCACUU CAACUCUGGUC(dT)(dT)-3′; siSET-2, sense 5′-GCGAUUGAACACAUUGAUGAA(dT)(dT)-3′, antisense 5′-UUCAUCAAUGUGUUCAAUCGC(dT)(dT)-3′. siCKAP4-1, sense 5′-GAAG UACCCUUCAGACUAU(dT)(dT)-3′, antisense 5′-AUAGUCUGAAGGGUACU UC(dT)(dT)-3′; siCKAP4-2, sense 5′-CAACGAGAACAAUCUG GAA(dT)(dT)-3′, antisense 5′-UUCCAGAUUGUUCUCGUUG(dT)(dT)-3′. siRAP1B-1, sense 5′-CGAUUUACAAGACCUGAGA(dT)(dT)-3′, antisense 5′-UCUCAGGUCUU GUAAAUCG(dT)(dT)-3′; siRAP1B-2, sense 5′-GAACAACUGUGCAUUCUUA (dT)(dT)-3′, antisense 5′-UAAGAAUGCACAGUUGUU C(dT)(dT)-3′. siPISD-1, sense 5′-CGUCGUGUGACUCCUUCAA (dT)(dT)-3′, antisense 5′-UUGAA GGAGUCACACGACG(dT)(dT)-3′; siPISD-2, sense 5′-CGGCAGUAUGAGAA GUACA(dT)(dT)-3′, antisense 5′-UGUACUUCUCA UACUGCCG(dT)(dT)-3′.

For PLIN3 overexpression, the coding sequence (CDS) of PLIN3 (Youbao Biotechnology, China) was cloned into the pLV3-CMV-MCS-3xHA-CopGFP-Puro vector for expression.

### Construction of PLIN3-knockdown and PLIN3-overexpression stable cell lines

To generate PLIN3-knockdown and PLIN3-overexpression stable HCC cell lines, we employed a lentivirus-mediated approach. For knockdown, a short hairpin RNA (shRNA) targeting PLIN3 (5′-GCCTGATGGAAACTGTCAAGC-3′) was designed using Sigma–Aldrich online tools and cloned into the pLKO.1 vector. For overexpression, a previously constructed pLV3-PLIN3 expression plasmid was used. Lentiviral particles were produced by co-transfecting HEK293T packaging cells with the respective lentiviral plasmid (10 μg), the psPAX2 packaging plasmid (5 μg; Addgene, #12260), and the pMD2.G envelope plasmid (7.5 μg; Addgene, #12259) using Lipofectamine 3000 (Invitrogen). At 48 h post-transfection, viral supernatant was collected, filtered through a 0.45-μm membrane, and used to transduce HCC cells in the presence of 8 μg/mL polybrene. Transduced cells were then selected with puromycin for 7–14 days, after which stable polyclonal populations were maintained in medium supplemented with 1 μg/mL puromycin.

### Cell culture

The human liver cancer cell lines MHCC97H and HLE were obtained from the American Type Culture Collection (ATCC), HCCLM3 cells were obtained from the National Collection of Authenticated Cell Cultures, and the PLC/PRF/5, SK-Hep1, HepG2, Huh7, and human embryonic kidney 293T (HEK293T) cell lines were obtained from Guangzhou Jennio Biotech Co., Ltd. The cells were cultured in Dulbecco’s Modified Eagle Medium (DMEM) supplemented with 10% fetal bovine serum and 1% penicillin/streptomycin, maintained at 37 °C in a humidified atmosphere containing 5% CO_2_. The cell lines underwent authentication through short tandem repeat (STR) analysis, were routinely screened for mycoplasma contamination, and were cultured for up to 20 passages (approximately two months) to ensure experimental consistency.

### Orthotopic xenograft models of HCC

Male BALB/C nude mice (5–6 weeks old) were obtained from Beijing Huafukang Biotech (Beijing, China). For orthotopic tumor implantation, 2 × 10^6^ PLIN3-knockdown or PLIN3-overexpression HCC cells or their parental counterparts were suspended in 20 μL of serum-free medium and combined with an equal volume (1:1 v/v) of Matrigel (BD Biosciences, USA). This cell–matrix mixture was subsequently injected into the left hepatic lobe of the nude mice. Tumor progression was systematically monitored, and the mice were humanely euthanized 8–10 weeks following implantation. Tumor volume was calculated using the formula: Tumor volume (mm^3^) = 0.5 × tumor length (mm) × tumor width (mm)^2^. Post-euthanasia, liver and lung tumor tissues were harvested and subjected to hematoxylin-eosin staining for histopathological analysis.

### LD isolation

LD isolation was performed following an established protocol. Briefly, cells treated with oleic acid (OA) for 24 h were washed twice with ice-cold phosphate-buffered saline (PBS), scraped, and resuspended in PBS. The cell suspension was centrifuged at 1000 *g* at 4 °C for 10 min to pellet the cells. The pellet was subsequently resuspended in ice-cold Buffer A (20 mM tricine (A600546, Sangon Biotech), pH 7.8, 250 mM sucrose (A420074, Sangon Biotech) supplemented with 0.2 mM PMSF (A100754, Sangon Biotech)) and incubated on ice for 20 min. Cell disruption was achieved using a nitrogen bomb (Parr Instrument, USA) at a pressure of 35 bar for 15 min on ice to release LDs from the cytoplasm. The resulting homogenate was centrifuged at 3000 *g* at 4 °C for 10 min to remove nuclei, cellular debris, and unbroken cells, yielding the post-nuclear supernatant. The post-nuclear supernatant was transferred to a centrifuge tube, overlaid with Buffer B (20 mM HEPES, pH 7.4, 100 mM KCl, and 2 mM MgCl_2_), and subjected to ultracentrifugation at 182,000 *g* at 4 °C for 1 h. Following centrifugation, the white band containing purified LDs at the top of the gradient was carefully collected. To remove residual contamination, the LD fraction was centrifuged at 20,000 *g* at 4 °C for 5 min, and the resulting pellet was resuspended in 200 μL of Buffer B to obtain a clean LD preparation. To assess purification efficiency, 1 mL of solution was collected from the middle of the gradient and centrifuged at 270000 *g* at 4 °C for 1 h. A 500 μL aliquot of the supernatant was retained as the cytosolic fraction (Cyt). Additionally, the pellet remaining in the centrifuge tube was washed three times with 1 mL of Buffer B and resuspended in 1 mL of Buffer B, representing the total membrane fraction. Finally, the purity of isolated LD proteins was evaluated by Western blotting, using concurrently prepared post-nuclear supernatant, cytosolic (Cyt), and total membrane fractions as controls.

### Cell transfection experiments

HCC cells were transfected *in vitro* utilizing Lipofectamine™ 3000 (Thermo Fisher Scientific, USA). siRNAs demonstrating significant knockdown efficiency and plasmids with potential for overexpression were selected for subsequent functional assays.

### Triglyceride detection assay

The intracellular triglyceride (TG) levels within the cells were quantified using a commercial triglyceride quantification kit (BC0625, Solarbio). In brief, 2 × 10^6^ cells were harvested, and 1 mL of extraction reagent was subsequently added. The samples were subjected to sonication for 1 min and then centrifuged at 8000 *g* at 4 °C for 10 min. The resulting supernatant was analyzed for TG content in accordance with the manufacturer’s instructions. TG concentrations were determined by referencing a standard curve.

### Cell proliferation assay

The proliferation of HCC cells was evaluated using the Cell Counting Kit-8 (CCK-8) assay (HY–K0301, MCE). Cells were seeded at a density of 2000–3000 cells per well in 96-well plates and cultured for 24–96 h. Subsequently, 10 μL of CCK-8 solution was added to each well containing 100 μL of medium, followed by an incubation period of 2 h at 37 °C. Absorbance was measured at 450 nm using an Epoch microplate reader (BioTek).

### Transwell assay

HCC cells were pre-treated with 5 μg/mL mitomycin C for 2 h and then seeded (10–20 × 10^4^ cells) into the upper chamber of a Transwell insert with an 8-μm pore size, excluding Matrigel. The lower chamber contained a full growth medium. Cells were incubated at 37 °C for 16–24 h, and then fixed with 4% paraformaldehyde and stained using 0.1% crystal violet. The membranes were mounted on slides, and migrated cells were counted under a microscope.

### Wound healing assay

HCC cells were cultured in 6-well plates until they reached approximately 80% confluence. The cells were then treated with mitomycin C for a duration of 2 h prior to creating a scratch using a pipette tip. Following the scratch, the cells were washed and incubated in a complete medium. Phase-contrast microscopy was employed to capture images at 0 and 48 h post-scratch. The migration rate was determined by measuring the width of the wound.

### Sorafenib sensitivity assay

For the cell line-based sorafenib sensitivity assays, the cells were seeded into 96-well plates at an appropriate density. After 12 h, drugs were added at the indicated concentrations (0–40 uM) using an 8-channel pipette (Eppendorf). The cells were incubated for 48 h, and cell proliferation was measured using the CCK-8 reagent. The absorbance was read at 450 nm using a microplate reader (Epoch, BioTek), which reflects the cell viability based on absorbance values.

### Immunofluorescence

HCC cells were cultured on glass slides and subsequently fixed with 4% paraformaldehyde. The cells were permeabilized using 0.5% Triton X-100 and treated with a blocking solution. Primary antibodies were applied to the cells and incubated at 4 °C overnight, followed by incubation with secondary antibodies at room temperature for 1 h. DAPI was utilized for nuclear counterstaining. The slides were mounted and analyzed using a Leica confocal microscope (Germany).

### BODIPY staining

HCC cells cultured on glass slides were fixed with 4% paraformaldehyde. Post-fixation, the cells were incubated with 2 μM BODIPY dye (D3922, Thermo) at room temperature for 15–30 min. Excess dye was removed by washing with PBS. The cells were imaged using a confocal microscope with an excitation wavelength of approximately 488 nm and an emission range of 510–550 nm.

### F-actin staining

F-actin staining was performed on HCC cells cultured on slides within 12-well plates. Following treatment, the cells were subjected to three washes with PBS, fixed with 4% paraformaldehyde for 15 min, and permeabilized using 0.5% Triton X-100 for 10 min. F-actin was stained using DyLight™ 488 Phalloidin at a 1:40 dilution at room temperature for 10 min. Nuclei were counterstained with DAPI in the dark for 20 min. Slides were mounted with an anti-fluorescent quenching agent to prevent fluorescence loss. Images were captured using a Leica confocal microscope (Wetzlar, Germany). ImageJ software was employed to quantify lamellipodia formation.

### Statistical analysis

Statistical analyses were conducted using GraphPad Prism software (version 8.0), with a *P*-value of less than 0.05 considered statistically significant. Unless otherwise indicated, data were presented as mean ± standard deviation from at least three independent experiments. The Mann–Whitney *U* test was used for comparisons between two independent groups, while the paired *t*-test assessed expression levels between HCC tissues and adjacent noncancerous tissues. For nonparametric comparisons between two groups, the Wilcoxon test was applied. Analysis of variance (ANOVA) was conducted to evaluate differences among multiple groups. Kaplan–Meier survival curves were generated, and their statistical significance was assessed using the log-rank test. Pearson’s correlation test was employed to examine the relationship between two variables.

## Results

### Molecular subtypes in HCC stratified by LDAGs

To systematically characterize LDAGs in HCC, we identified 122 LDAGs through curation of the AmiGO2 database via the “lipid droplet” ontology term (Fig. S1A; Table S1). Protein–protein interaction network analysis integrating BioGRID interaction data with UniProt functional annotations demonstrated functional clustering corresponding to core biological processes. Metabolic pathways constituted the predominant functional category, with over half of the LDAGs enriched in triacylglycerol metabolism, sterol biosynthesis, retinol homeostasis, and redox regulation (Fig. 1A; Table S2). The nonmetabolic modules included vesicle trafficking, cytoskeletal dynamics, transmembrane transport, and programmed cell death pathways (Fig. 1A; Table S2). Pathway analyses (GO/KEGG) further revealed LDAG involvement in the Apelin, AMPK, and NFκB signaling cascades (Fig. S1B). We conducted a multicohort expression analysis on 1834 HCC tumors and 1169 matched non-tumor tissues, utilizing data from the TCGA-LIHC, ICGC-LIRI-JP, and seven GEO datasets (GSE25097, GSE102083, GSE36376, GSE112790, GSE45436, GSE124535, GSE14520) (Table S3). Despite the inherent limitations of microarray platforms in detecting low-abundance transcripts, most LDAGs presented significant tumor-specific up-regulation in HCC (Fig. 1B; Fig. S1C–S1E; Table S4).

Subsequently, we employed unsupervised consensus clustering analysis based on the expression patterns of 122 LDAGs. HCC patients within the TCGA-LIHC cohort were categorized into three subgroups, labeled as C1–C3 subtypes (Fig. 1C; Fig. S2A–S2C; Table S5). These subgroups represented high (C1), intermediate (C2), and low (C3) LDAG expression levels, respectively (Fig. 1D). Notably, the C1 subgroup demonstrated advanced tumor stages, enhanced inflammatory infiltration, poor histological differentiation grades, and the shortest median overall survival (Fig. 1E and F; Fig. S2D).

The existence and robustness of these LDAG subtypes were confirmed using three independent datasets: ICGC-LIRI-JP, GSE116174, and GSE14520 (Fig. S2E–S2P). In line with the distinct clinical sample categorization, the LDAG classification was applied to HCC cell lines from the CCLE datasets. Cell lines like Huh7 and PLC/PRF/5 are categorized as C1 subtypes, while SNU475 and SNU761 fall under the C3 subtypes (Fig. S2Q). These findings indicate that LDAG classification is consistently stable across different datasets and correlates with clinicopathologic indices that have prognostic significance.

Research has identified various molecular classifications of HCC, including the classification by Li et al,^29^ Jiang et al,^30^ Gao et al,^8^ Chiang et al,^31^ Boyault et al,^32^ Lee et al,^33^ Coulouarn et al,^34^ and Liu et al.^35^ The comparison of LDAG subtypes with existing HCC classifications showed significant overlap (Fig. 1G), indicating the reliability of the LDAG clustering process.

### Genomic and signaling landscape of LDAG subtypes in HCC

Mutations in tumor driver genes, such as TP53 and CTNNB1, have been strongly associated with poor prognosis in cancer patients 36, 37, 38, 39. We analyzed the genomic characteristics of LDAG subtypes within the TCGA-LIHC cohort. The C1 subtype exhibited the highest TP53 mutational ratio while the C3 subtype showed the highest CTNNB1 mutational ratio (Fig. 2A and B; Fig. S3A–S3C).

We then investigated the signaling characteristics across LDAG subtypes. Cluster-specific DEGs were identified with criteria |log_2_FC| ≥ 1 and *P* < 0.05, resulting in 1438 DEGs for C1 (1168 up-regulated, 270 down-regulated), 397 DEGs for C2 (302 up-regulated, 95 down-regulated), and 406 DEGs for C3 (13 up-regulated, 393 down-regulated) (Fig. 2C; Table S6). Pathway enrichment analysis identified unique molecular signatures for each of the three LDAG subtypes. The C1 subtype exhibited pronounced proliferative signaling (*e.g.*, motor proteins, tight junctions, and the cell cycle), whereas the C2 subtype was enriched in metabolic pathways (*e.g.*, tryptophan catabolism and bile acid biosynthesis). In contrast, the C3 subtype showed concurrent activation of xenobiotic metabolism (cytochrome P450) and immune modulation (cytokine–receptor interactions) (Fig. 2D; Fig. S3D‒S3F; Table S7).

GSVA-based hallmark gene set analysis highlighted distinct profiles: subtype C1 exhibited proliferation-driven processes (mitotic spindle, Wnt/β-catenin, MYC targets, and G2M checkpoint), subtype C2 demonstrated a predominance of metabolic processes (fatty acid and bile acid metabolism), whereas subtype C3 presented dual activation in immune pathways (coagulation, complement, and IL6-JAK-STAT3) and metabolic pathways (xenobiotic metabolism) (Fig. 2E; Fig. S3G–S3I; Table S7). LDAG subtypes constitute distinct patient groups characterized by unique genomic and molecular traits, which may be susceptible to current therapeutic agents.

### Therapeutic vulnerabilities in the LDAG subtypes for HCC

HCC classification offers a crucial understanding of its molecular diversity, potentially informing personalized treatment strategies.^7^ To delineate subtype-specific vulnerabilities within the LDAG-based classification framework, we focused on the C1 subtype, which has the poorest clinical prognosis. We first started with preclinical drug discovery via the oncoPredict algorithm, which integrates pharmacogenomic data from GDSC, CTRP, and PRISM. Twenty-two candidate compounds with predicted C1-specific efficacy were identified (Fig. 3A and B; Table S8). Notably, key therapeutic targets, including TOP2A (doxorubicin), AURKA (alisertib), and PDGFRB/FGFR4/FLT1 (nintedanib), were significantly overexpressed in C1 tumors (Fig. 3C; Table S9). These data suggest that the C1 subtype has molecular dependencies that targeted therapeutic strategies can exploit.

Furthermore, we analyzed the association between the LDAG subtypes and response to therapeutic interventions in HCC. Patients treated with sorafenib were stratified according to their LDAG subtype (Fig. 3D). Notably, patients in the C1 subtype exhibited the highest objective response rate to sorafenib, whereas a majority (95%) of those in the C2 subtype showed no clinical response (Fig. 3E). To validate these clinical observations, we utilized the CCLE dataset, in which HCC cell lines had previously been classified using the LDAG framework (Fig. S2Q). Cell lines representative of the C1 and C2 subtypes were selected for *in vitro* validation. Consistent with the patient-derived data, Huh7, PLC/PRF/5, and HepG2 cell lines (C1 subtype) demonstrated relative sensitivity to sorafenib. In contrast, HLE and SK-Hep-1 cell lines (C2 subtype) displayed relative resistance to the treatment (Fig. 3F).

Next, we investigated potential associations between LDAG subtypes and treatment outcomes for trans-arterial chemoembolization as well as immunotherapy responsiveness using publicly available datasets. Analysis of the GSE104580 cohort revealed that the C1 subtype was associated with the poorest response rate to trans-arterial chemoembolization, with 76% of C1 patients classified as non-responders (Fig. 3G and H). Analysis of an independent Korean cohort comprising 40 HCC patients treated with anti-PD-1 therapy revealed that the C1 subtype was associated with a marginally elevated response rate relative to other subtypes. However, it should be noted that the response rates to immunotherapy remained generally limited across all three LDAG subtypes (Fig. 3I and J). Collectively, these findings indicate that the LDAG C1 subtype exhibits enhanced sensitivity to sorafenib therapy.

### Machine learning-based integration constructs a prognostic model for HCC using hub LDAGs

**Figure 4:**
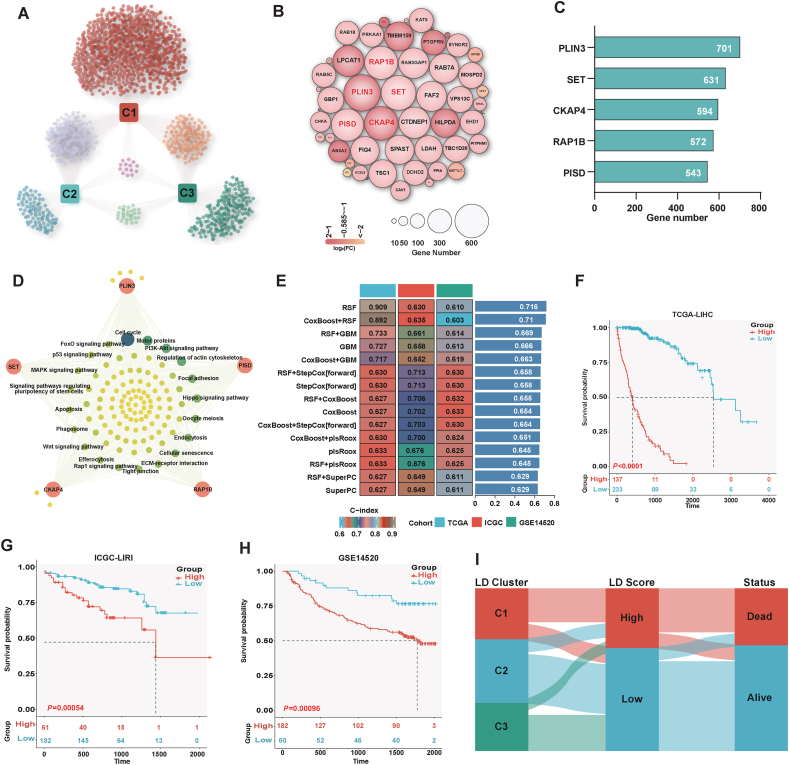
Characterization and prognostic analysis of hub lipid droplet-associated genes (LDAGs) in hepatocellular carcinoma**. (A)** Regulatory network between LD-differentially expressed genes (DEGs) and non-LD-DEGs across the three clusters. **(B)** Correlation analysis between LDAGs and non-LD-DEGs in the C1 subtype. The size of each circle represents the number of non-LD-DEGs highly correlated with the corresponding LDAG. The intensity of the red color indicates the differential expression of LDAGs between the C1 subtype and the other subtypes, with red representing the top five most significant LDAGs. **(C)** The number of hallmark genes and non-LD-DEGs is highly correlated with the corresponding hub LDAGs. **(D)** Pathway enrichment analysis of non-LD-DEGs highly correlated with hub LDAGs (PLIN3, SET, CKAP4, RAP1B, and PISD) in the C1 subtype. **(E)** A combination of machine learning predictive models calculates the C-index for each model on the training set and the validation set. **(F–H)** Kaplan–Meier curves of OS in the Train (TCGA-LIHC) and test sets (ICGC-LIRI-JP and GSE14520) based on the model showed longer survival time in low-risk groups. **(I)** Sankey diagram illustrating the relationships between molecular subtypes, risk groups, and patient outcomes.

To identify LDAGs critically involved in HCC pathogenesis, we performed correlation analyses between LD-related DEGs (LD-DEGs) and non-LD-DEGs across three molecular subtypes (Table S10). C1 subtype exhibited the strongest trans-regulatory potential over non-LD-DEG networks (Fig. 4A). Further investigation of the LD-DEGs in C1 subtype revealed five hub genes (PLIN3, SET, CKAP4, RAP1B, and PISD) that had co-expression relationships with more than 500 non-LD-DEGs (Fig. 4B and C). Functional enrichment analysis revealed their significant role in oncogenic pathways essential for tumor progression, such as the cell cycle, motor proteins, and the PI3K–AKT signaling pathway (Fig. 4D; Table S11).

Furthermore, an integrative approach utilizing machine learning was employed to analyze the five genes, culminating in the identification of a consensus LDAG signature for predicting overall survival in HCC patients. The leave-one-out cross-validation (LOOCV) framework was applied to fit a combination of ten machine learning algorithms, with hyperparameter tuning conducted on the training set. Subsequently, the concordance index (C-index) for each model was calculated on the testing set (Fig. 4E). In the TCGA-LIHC training dataset, as well as in two validation datasets (ICGC-LIRI-JP and GSE14520), patients classified as high-risk exhibited significantly reduced overall survival compared with those in the low-risk group (Fig. 4F–H). A Sankey diagram illustrates the relationships between LDAG-based molecular subtypes, hub LDAG risk scores, and clinical outcomes (Fig. 4I). The findings indicated that the LDAG signature effectively predicted prognosis in HCC patients.

### The expression of PLIN3 is elevated in HCC and is correlated with poor prognosis

**Figure 5:**
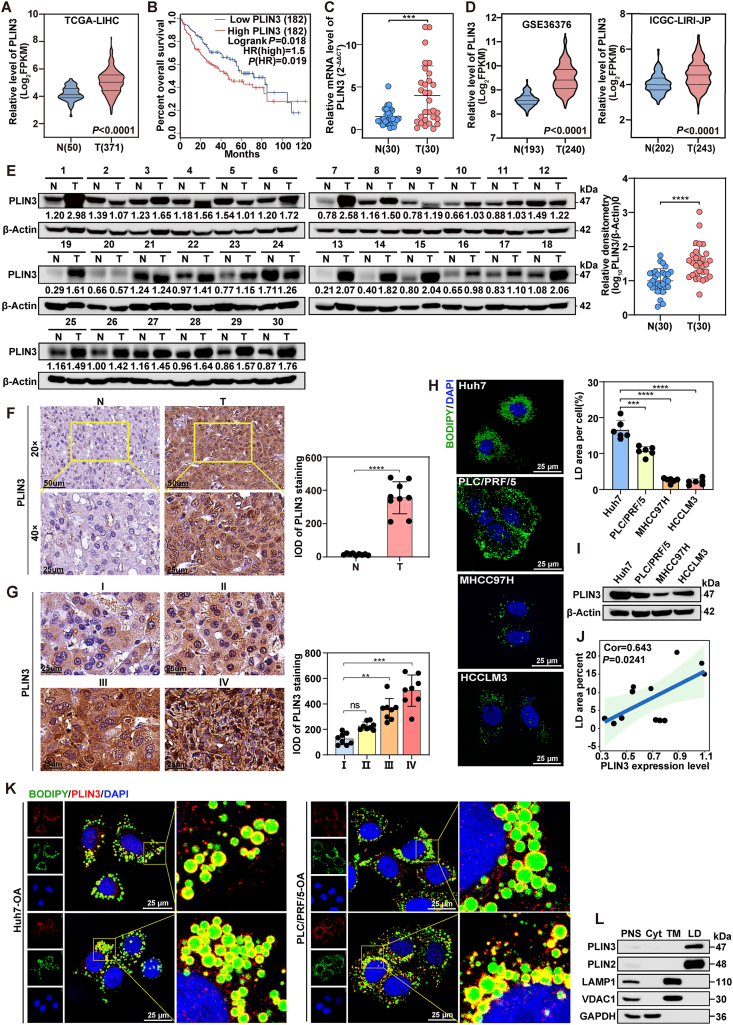
Up-regulation of PLIN3 in hepatocellular carcinoma (HCC) and its association with poor prognosis. **(A)** Expression levels of PLIN3 in HCC tissues (T) compared with non-tumor tissues (N), based on data from the TCGA-LIHC cohort. **(B)** Association between PLIN3 expression and overall survival (OS) in HCC patients, based on data from the TCGA-LIHC cohort. **(C)** mRNA level of PLIN3 in 30 HCC tissue samples (T) and paired non-tumor tissues (N) detected by quantitative PCR. **(D)** Expression levels of PLIN3 in HCC tissues compared with non-tumor tissues were analyzed in the GSE36376 and ICGC-LIRI-JP datasets. **(E)** Protein levels of PLIN3 in 30 HCC tissue samples (T) and paired non-tumor tissues (N) detected by Western blotting. **(F)** Immunohistochemical analysis of PLIN3 expression in non-tumor versus HCC tissues. **(G)** Immunohistochemical staining shows the intensity of PLIN3 expression across different tumor stages in HCC tissues. **(H)** BODIPY staining of HCC cell lines to assess LD accumulation. **(I)** Western blotting analysis of PLIN3 expression in various HCC cell lines. **(J)** Correlation between PLIN3 protein abundance and BODIPY staining intensity across four HCC cell lines was analyzed. **(K)** Immunofluorescence co-staining of PLIN3 and LDs was performed in Huh7 and PLC/PRF/5 cells following OA stimulation. **(L)** The enrichment of PLIN3 in isolated LD proteins from OA-induced Huh7 cells was evaluated by Western blotting, using post-nuclear supernatant (PNS), cytoplasmic fraction (Cyt), and total membrane fraction (TM) as controls. ∗*P* < 0.05, ∗∗*P* < 0.01, ∗∗∗*P* < 0.001, and ∗∗∗∗*P* < 0.0001.

We first evaluated the clinical relevance of five hub LDAGs (PLIN3, SET, CKAP4, RAP1B, and PISD) using the TCGA-LIHC dataset. All five genes were significantly overexpressed in HCC tissues compared with adjacent non-tumoral liver tissues (Fig. 5A; Fig. S4A). Notably, elevated mRNA levels of PLIN3, SET, CKAP4, and RAP1B were associated with reduced overall survival (Fig. 5B; Fig. S4B). Validation in an independent cohort of 30 matched HCC/non-tumoral tissue pairs confirmed the tumor-specific up-regulation of PLIN3, SET, and CKAP4 (Fig. 5C; Fig. S4C). Among these genes, PLIN3 demonstrated the most pronounced differential expression, identifying it as a primary candidate for further investigation.

To evaluate its clinical relevance, we analyzed PLIN3 expression across multiple independent cohorts (GSE36376, GSE45436, GSE112790, and ICGC-LIRI-JP). PLIN3 was consistently up-regulated in HCC tissues (Fig. 5D; Fig. S4D). High PLIN3 expression correlated significantly with aggressive clinicopathological features, including advanced TNM stage, poor histological differentiation, and an increased incidence of lung metastasis (Fig. S4E). Furthermore, multivariate Cox regression analysis identified high PLIN3 expression as an independent prognostic factor for poor patient outcomes (Fig. S4F). We next evaluated the PLIN3 protein level in 30 matched pairs of HCC and adjacent non-tumorous tissues. Consistent with transcriptional data, PLIN3 protein levels were significantly elevated in tumor tissues (Fig. 5E), a finding corroborated by immunohistochemistry (Fig. 5F). Moreover, PLIN3 protein expression exhibited a progressive increase corresponding to more advanced HCC stages (Fig. 5G).

To functionally link PLIN3 to lipid metabolism in HCC, we performed BODIPY staining in four HCC cell lines, revealing a strong positive correlation between PLIN3 protein abundance and intracellular LD content (Fig. 5H–J). Immunofluorescence analysis in Huh7 and PLC/PRF/5 cells treated with OA to induce LD biogenesis showed extensive colocalization of PLIN3 with LDs (Fig. 5K). Furthermore, Western blotting analysis of subcellular fractions confirmed a substantial enrichment of PLIN3 in the purified LD fraction from OA-induced Huh7 cells (Fig. 5L). These results demonstrate that PLIN3, a canonical LD-associated protein, is consistently overexpressed in HCC. Its expression correlates with aggressive tumor features, poor survival, and LD abundance, establishing PLIN3 as a promising prognostic biomarker for HCC.

### Silencing of PLIN3 inhibits HCC proliferation and metastasis

**Figure 6:**
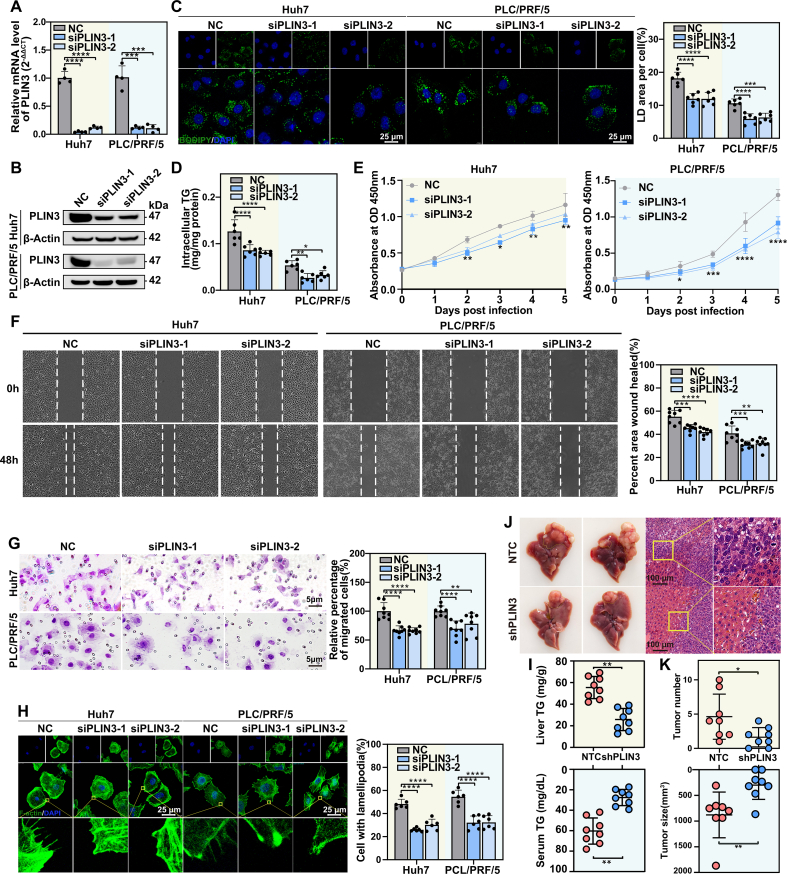
PLIN3 knockdown inhibits hepatocellular carcinoma cell proliferation and migration. **(A, B)** Quantitative PCR and Western blotting analyses of PLIN3 mRNA and protein expression in PLIN3-knockdown Huh7 and PLC/PRF/5 cells, respectively. **(C)** BODIPY staining was used to assess LD accumulation in PLIN3-knockdown Huh7 and PLC/PRF/5 cells. **(D)** Measurement of TG content in PLIN3-knockdown Huh7 and PLC/PRF/5 cells. **(E)** Cell proliferation was assessed via a CCK-8 assay to evaluate the effect of PLIN3 knockdown on Huh7 and PLC/PRF/5 cells. **(F, G)** Wound healing and migration assays were performed to assess the migration of PLIN3-knockdown Huh7 and PLC/PRF/5 cells. **(H)** F-actin staining of PLIN3-knockdown Huh7 and PLC/PRF/5 cells was performed to examine cytoskeletal changes. **(I)** Serum and hepatic TG levels from orthotopic xenografts. **(J)** Representative images of liver tissues and hematoxylin-eosin-stained tumor sections from orthotopic xenografts. **(K)** Quantitative analysis of the orthotopic liver tumor burden. All the data are presented as the means ± standard deviation. Statistical significance is indicated as ∗*P* < 0.05, ∗∗*P* < 0.01, ∗∗∗*P* < 0.001, and ∗∗∗∗*P* < 0.0001.

To elucidate the functional role of PLIN3 in HCC, we performed siRNA-mediated knockdown of PLIN3 in two LD-rich HCC cell lines, Huh7 and PLC/PRF/5 (Fig. 6A and B). Silencing of PLIN3 resulted in a significant reduction in lipid accumulation, as reflected by reduced LD content and decreased TG levels (Fig. 6C and D). Functionally, PLIN3 knockdown impaired cellular proliferation, as measured by CCK-8 assays (Fig. 6E), and attenuated migratory capacity, as demonstrated by wound healing and Transwell migration assays (Fig. 6F and G). Furthermore, cytoskeletal analysis via F-actin staining indicated that PLIN3 depletion suppressed lamellipodia formation, consistent with a less invasive phenotype (Fig. 6H). Notably, OA treatment significantly exacerbated the inhibitory effects of PLIN3 knockdown on both cell proliferation and migration (Fig. S5).

To validate these findings *in vivo*, orthotopic xenograft models were established by injecting control or PLIN3-knockdown Huh7 cells into the liver lobes of BALB/c nude mice. PLIN3 depletion led to significant reductions in both serum and hepatic TG levels, along with a marked decrease in tumor number and size within the liver (Fig. 6I–K). Together, these *in vitro* and *in vivo* results establish PLIN3 as a critical regulator of HCC growth.

Concurrently, we performed preliminary functional characterization of the remaining four key LDAGs (SET, CKAP4, RAP1B, and PISD) using CCK-8 proliferation assays and migration experiments. siRNA-mediated knockdown of these genes consistently suppressed both proliferative and migratory capacities of HCC cells (Fig. S6). These results imply that all five identified LDAGs are likely involved in promoting HCC, although further experimental validation is required to fully substantiate these observations.

### PLIN3 overexpression promotes HCC proliferation and metastasis

**Figure 7:**
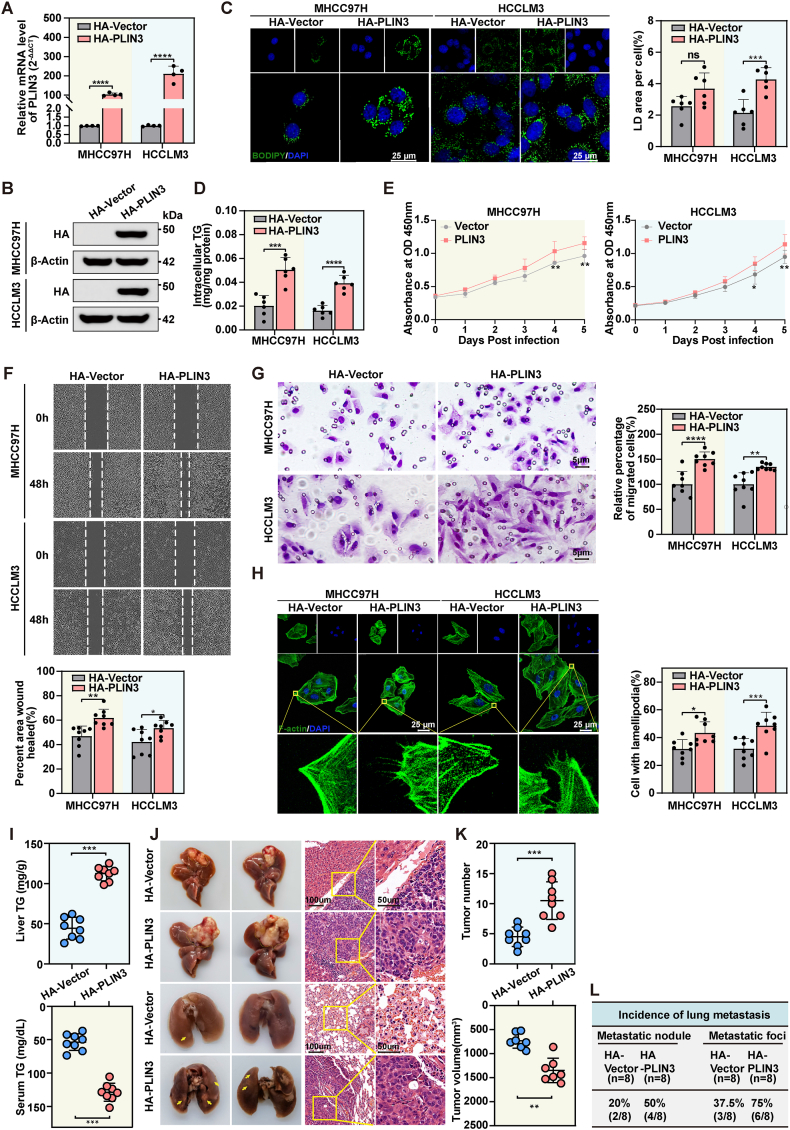
PLIN3 overexpression increases hepatocellular carcinoma cell proliferation and migration. **(A, B)** Quantitative PCR and Western blotting analyses of PLIN3 mRNA and protein levels in PLIN3-overexpressing MHCC97H and HCCLM3 cells, respectively. **(C)** BODIPY staining was used to assess LD accumulation in PLIN3-overexpressing MHCC97H and HCCLM3 cells. **(D)** Measurement of TG content in PLIN3-overexpressing MHCC97H and HCCLM3 cells. **(E)** Cell proliferation was assessed via a CCK-8 assay to evaluate the effect of PLIN3 overexpression on MHCC97H and HCCLM3 cell growth. **(F, G)** Wound healing and migration assays were performed to assess the migration of PLIN3-overexpressing MHCC97H and HCCLM3 cells. **(H)** F-actin staining was used to examine cytoskeletal alterations in PLIN3-overexpressing MHCC97H and HCCLM3 cells. **(I)** Serum and hepatic TG levels were measured in orthotopic xenograft models. **(J)** Representative images of liver tissues and corresponding hematoxylin-eosin-stained tumor sections from orthotopic xenografts are shown. **(K, L)** Quantitative analyses of orthotopic liver tumor burden and lung metastasis incidence. All the data were presented as mean ± standard deviation. Statistical significance is indicated as ∗*P* < 0.05, ∗∗*P* < 0.01, ∗∗∗*P* < 0.001, and ∗∗∗∗*P* < 0.0001.

To further investigate the oncogenic function of PLIN3 in HCC, PLIN3 was ectopically expressed in two HCC cell lines (MHCC97H and HCCLM3) with intrinsically low LD content (Fig. 7A and B). Overexpression of PLIN3 significantly enhanced lipid accumulation, as evidenced by elevated LD quantity and increased TG levels (Fig. 7C and D). Subsequent functional assays demonstrated that PLIN3 overexpression promoted HCC cell proliferation (CCK-8 assay) and stimulated migratory capacity (wound healing and Transwell assays) (Fig. 7E–G). Consistent with a more aggressive phenotype, cytoskeletal analysis via F-actin staining revealed that PLIN3 overexpression facilitated lamellipodia formation (Fig. 7H). OA treatment synergistically amplified the pro-proliferative and pro-migratory effects induced by PLIN3 overexpression in these cells (Fig. S7).

To evaluate the *in vivo* tumor-promoting role of PLIN3, an orthotopic pulmonary metastasis HCC mouse model was established by injecting control or PLIN3-overexpressing MHCC97H cells into the liver lobes of BALB/c nude mice. PLIN3 overexpression led to significantly elevated serum and hepatic TG levels (Fig. 7I), and markedly enhanced tumorigenesis, as reflected by increased tumor number and size within the liver (Fig. 7J and K). Moreover, mice injected with PLIN3-overexpressing cells exhibited a significant increase in pulmonary metastatic nodules (Fig. 7L). Collectively, these *in vitro* and *in vivo* findings indicate that PLIN3 overexpression drives lipid accumulation and promotes HCC proliferation and metastasis.

### Transcriptomic and pathway analyses reveal mechanisms downstream of PLIN3

**Figure 8:**
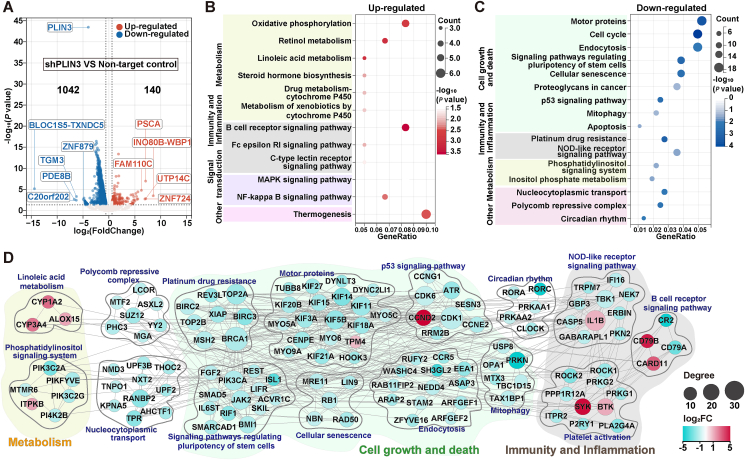
Differentially expressed genes (DEGs) and functional enrichment analysis in PLIN3-knockdown versus control Huh7 cells. **(A)** Identification of DEGs between PLIN3-knockdown and control Huh7 cells. **(B, C)** Functional enrichment analyses of upregulated and downregulated DEGs, respectively. **(D)** Protein–protein interaction (PPI) networks of the identified DEGs.

To elucidate the molecular mechanisms through which PLIN3 drives HCC, we performed RNA-seq on PLIN3-knockdown and control Huh7 cells. Differential expression analysis identified 1182 significantly altered mRNAs, including 140 up-regulated and 1042 down-regulated transcripts (Fig. 8A; Table S12). KEGG pathway enrichment analysis revealed that genes up-regulated upon PLIN3 knockdown were primarily enriched in metabolic pathways such as oxidative phosphorylation, cytochrome P450 drug metabolism, and linoleic acid metabolism (Fig. 8B). Conversely, down-regulated genes were significantly associated with processes related to cell growth and death, such as motor proteins, cell cycle, signaling pathways regulating pluripotency of stem cells, and cellular senescence (Fig. 8C; Table S13).

To further explore functional interactions among the DEGs, we integrated the RNA-seq data with the STRING database to construct protein–protein interaction networks. This analysis highlighted prominent alterations in genes belonging to several key functional categories, specially signaling pathways regulating stem cell pluripotency (*e.g.*, PIK3CA, JAK2, SMAD5, FGF2, BMI1), motor proteins (*e.g.*, KIF3A, KIF5B, KIF14, MYO5A, MYO9A), linoleic acid metabolism (*e.g.*, CYP1A2, CYP3A4, ALOX15), and NOD-like receptor signaling pathways (*e.g.*, IL1B, TRPM7, ERBIN) (Fig. 8D). These molecular changes collectively support the phenotypic alterations observed in PLIN3-knockdown HCC cells.

We further validated these findings using transcriptomic data from the TCGA-LIHC cohort. HCC patients were stratified into PLIN3-high and PLIN3-low groups based on PLIN3 expression levels (Fig. S8A; Table S14). Enrichment analysis of DEGs between these groups showed strong concordance with the pathways identified in our cellular model (Fig. S8B and S8C; Table S15). Correlation analyses revealed that PLIN3 expression exhibited positive associations with multiple indices of tumor progression, including markers of proliferation (*e.g.*, Ki-67), cell cycle progression, epithelial–mesenchymal transition, and cytoskeletal motor activity (Fig. S8D). These analyses reveal that PLIN3 modulates a network of genes involved in lipid metabolism, stemness, cytoskeletal dynamics, and proliferation-related signaling, thereby promoting HCC malignancy.

## Discussion

HCC is characterized by its heterogeneity and intricate molecular profile, which complicates both prognosis and treatment strategies. This heterogeneity is evident in the variability of clinical manifestations, tumor progression, and therapeutic responses, thereby contributing to the generally poor prognosis observed in patients with advanced-stage disease.^2^^,^^3^ Notably, HCC is fundamentally a metabolic disorder, with dysregulated lipid metabolism playing a pivotal role in its pathogenesis.^40^^,^^41^ LDs, once considered mere storage organelles, are now recognized as dynamic entities actively participating in cellular metabolism, energy regulation, signal transduction, and stress responses 10, 11, 12, 13. The altered dynamics of LDs in HCC, including lipid accumulation and remodeling, indicate that LDs may function as both markers and regulators of tumorigenesis.^10^^,^^13^

Over the years, molecular subtyping of HCC has emerged as a powerful tool for predicting patient outcomes and tailoring therapeutic approaches. Traditional HCC subtyping has largely relied on genetic mutations, histopathological features, and clinical parameters.^6^^,^^42^^,^^43^ Recent insights into cancer metabolic reprogramming have facilitated the creation of novel subtyping methods, such as those focusing on lipid metabolism.^44^^,^^45^ Subtyping HCC based on LDAGs provides valuable insights into tumor heterogeneity and offers a promising approach for stratifying patients according to their unique metabolic signatures.

This study categorizes HCC patients into three subgroups based on LDAG expression patterns. These subtypes reflect underlying metabolic alterations in HCC and significantly differ in terms of clinical outcomes and therapeutic targets. The C1 subtype demonstrated the most aggressive clinical characteristics, characterized by advanced disease stages, activation of cell proliferation and metastasis pathways, and notably poorer overall survival. Further investigation of the C1 subtype revealed potential therapeutic targets, with five hub LDAGs—PLIN3, SET, CKAP4, RAP1B, and PISD—showing a strong association with this aggressive subtype. The expression of these genes was strongly associated with patient outcomes, indicating their potential as prognostic biomarkers.

Among the LDAGs identified in our study, PLIN3 stood out as a critical regulator of HCC progression. Perilipins, including PLIN3, play crucial roles in LD formation, stability, and turnover and have been implicated in various cancers 46, 47, 48, 49. Previous research has suggested that the up-regulation of PLIN3 in various cancers is linked to poor prognosis 49, 50, 51, 52. Our findings corroborate the notion that PLIN3 is significantly overexpressed in HCC tissues and is associated with adverse clinical outcomes, such as advanced tumor stage, reduced survival rates, and metastasis. Furthermore, through multivariate Cox regression analysis, PLIN3 was identified as an independent prognostic indicator for HCC.

Functional experiments revealed that overexpressing PLIN3 in HCC cells led to dramatic increases in LD accumulation, cell proliferation, and migration. Our integration of these findings with existing research highlights PLIN3’s crucial role in LD biology, underscoring its potential as a prognostic biomarker and therapeutic target for HCC.

Our findings offer important insights into the role of LDs in HCC, but several limitations must be recognized. Our study utilized publicly available datasets and patient samples, which, despite being large and diverse, may not comprehensively represent the molecular complexity of all HCC subtypes. Our data indicate a strong association between PLIN3 and poor prognosis, but the causal mechanisms in HCC progression require further elucidation. Furthermore, while LD-based subtyping appears promising, its clinical application requires validation in independent, prospective cohorts to assess its utility in guiding treatment decisions.

In conclusion, our study highlights the significant role of LDs and LDAGs, particularly PLIN3, in the diagnosis and progression of HCC. Identifying distinct HCC molecular subtypes through LDAG expression offers new insights into metabolic heterogeneity and its link to clinical outcomes. PLIN3, an overexpressed protein in HCC, shows significant promise as a prognostic marker and therapeutic target. Our findings suggest that LDAG-based profiling could be integrated into existing diagnostic and prognostic frameworks to improve the stratification of HCC patients and inform personalized treatment strategies. Further validation and mechanistic investigations are necessary to substantiate these findings and explore the therapeutic implications of targeting LD metabolism in HCC.

## CRediT authorship contribution statement

**Huiying Gu:** Writing – original draft, Methodology, Funding acquisition, Data curation. **Qiumin Wu:** Methodology, Formal analysis, Data curation. **Haibei Zhao:** Methodology, Formal analysis, Data curation. **JingLong Du:** Software, Resources. **Zhenzhen Zhang:** Writing – review & editing, Supervision, Project administration, Conceptualization. **Juan Chen:** Writing – review & editing, Project administration, Funding acquisition, Conceptualization.

## Ethics declaration

This study complied with the guidelines of the Declaration of Helsinki and received approval from the Chongqing Medical University Ethics Committee (approval number: 2024028). Written informed consent was obtained from all participants before they participated in the study.

## Data availability

Transcriptomic data can be accessed from TCGA, ICGC, or GEO under the following identifiers: TCGA-LIHC, ICGC-LIRI-JP, GSE25097, GSE36376, GSE45436, GSE102083, GSE112790, GSE124535, and GSE14520. Additional data can be accessed upon reasonable request.

## Funding

This research was funded by grants from the 10.13039/501100012166National Key Research and Development Program of China (No. 2022YFA1303600) and the Natural Science Foundation Project of Chongqing, China (No. CSTB2022NSCQ-MSX1277).

## Conflict of interests

Juan Chen is an Editorial Board member of *Genes & Diseases* and was not involved in the editorial review or the decision to publish this article. The remaining authors declare that there are no competing interests.

## Appendix A Supplementary data

Multimedia component 1 Multimedia component 1

Figure S8 Differentially expressed genes (DEGs) and functional enrichment analysis between the low and high PLIN3 expression groups of hepatocellular carcinoma (HCC) patients from the TCGA-LIHC dataset. **(A)** Identification of DEGs between the low and high PLIN3 expression groups of HCC patients from the TCGA-LIHC dataset**. (B, C)** Functional enrichment analyses of upregulated and downregulated DEGs, respectively. **(D)** Correlation analysis between PLIN3 expression and key signaling pathway scores. Statistical significance is indicated as “ns” (no significant difference); ∗*P* < 0.05, ∗∗*P* < 0.01, ∗∗∗*P* < 0.001, and ∗∗∗∗*P* < 0.0001. Figure S8

Multimedia component 2 Multimedia component 2

Figure S1 Functional and expression analyses of lipid droplet-associated genes (LDAGs) in hepatocellular carcinoma (HCC)**. (A)** Illustration of the 122 LD-associated proteins identified via the AmiGO2 database. Squares represent the functional categories to which these LD proteins belong. **(B)** GO and KEGG pathway analyses of LD-associated proteins. **(C**–**E)** Heatmap showing the expression profiles of LDAGs in HCC tissues and non-tumor tissues, based on data from the TCGA-LIHC, ICGC-LIRI-JP, and GSE14520 datasets, respectively. Figure S1

Figure S2 Subtype and clustering analyses of lipid droplet-associated genes (LDAGs) in hepatocellular carcinoma (HCC). **(A)** Cumulative distribution function (CDF) plot of clustering results based on LDAG expression patterns. **(B)** Delta area of the CDF for clustering results based on LDAG expression patterns. **(C)** Principal component analysis (PCA) of LDAG expression in the HCC subgroups. **(D)** Association between the three identified HCC clusters based on LDAGs and various clinical features, including sex, age, BMI, TNM stage, histologic grade, inflammation, fibrosis, and survival status. **(E**–**G)** Heatmap of the consensus matrix based on the expression of 122 LDAGs in the ICGC-LIRI-JP, GSE14520, and GSE116174 cohorts. **(H**–**J)** PCA of LDAG expression patterns across the three HCC subgroups from the ICGC-LIRI-JP, GSE14520, and GSE116174 cohorts. **(K**–**M)** Heatmap showing the expression patterns of LDAGs across the three subgroups in the ICGC-LIRI-JP, GSE14520, and GSE116174 cohorts. **(N–P)** Kaplan–Meier survival analysis of the three subgroups based on the gene expression data of 122 LDAGs in the ICGC-LIRI-JP, GSE14520, and GSE116174 datasets. C1, Cluster 1; C2, Cluster 2; C3, Cluster 3. Statistical significance is indicated as ∗*P* < 0.05, ∗∗*P* < 0.01, ∗∗∗*P* < 0.001, and ∗∗∗∗*P* < 0.0001. Figure S2

Figure S3 Gene mutations and enrichment of pathways across the three-lipid droplet-associated genes (LDAGs)-associated hepatocellular carcinoma (HCC) subtypes. Gene mutation profiles of the three LDAG-associated HCC clusters. **(A**–**C)** Oncoplots showing the top 25 genes with somatic mutations in C1 (A), C2 (B), and C3 (C) subtypes. **(D**–**F)** Hyperactive and hypoactive processes of KEGG-enriched pathways in C1 (D), C2 (E), and C3 (F) subtypes. **(G**–**I)** Hyperactive and hypoactive processes of hallmark gene sets in C1 (G), C2 (H), and C3 (I) subtypes. Figure S3

Figure S4 Expression and prognostic relevance of hub lipid droplet-associated genes (LDAGs) in hepatocellular carcinoma (HCC). **(A)** Expression levels of the four hub LDAGs (SET, CKAP4, RAP1B, and PISD) in HCC tissues (T) compared with non-tumor tissues (N), as analyzed in the TCGA-LIHC dataset. **(B)** Association between the expression of the four hub LDAGs (SET, CKAP4, RAP1B, and PISD) and the OS rate in HCC patients, based on data from the TCGA-LIHC cohort. **(C)** mRNA levels of four hub LDAGs (SET, CKAP4, RAP1B, and PISD) in HCC tissue samples (T) and paired non-tumor tissues (N) were detected by quantitative PCR. **(D)** Expression levels of PLIN3 in HCC tissues compared with nontumor tissues were analyzed in the GSE45436 and GSE112790 datasets. **(E)** Analysis of the association between PLIN3 expression and various clinical features, including sex, BMI, TNM stage, histologic grade, inflammation, metastasis, and survival status, in HCC patients from the TCGA-LIHC dataset. **(F)** A nomogram was constructed based on a multivariate Cox regression analysis of PLIN3 expression and clinical characteristics to predict overall survival in HCC patients in terms of 1-year, 3-year, and 5-year survival. Statistical significance is indicated as “ns” (no significant difference); ∗*P* < 0.05, ∗∗*P* < 0.01, ∗∗∗*P* < 0.001, and ∗∗∗∗*P* < 0.0001. Figure S4

Figure S5 PLIN3 knockdown suppresses proliferation and migration of hepatocellular carcinoma cells under oleic acid (OA)-stimulated conditions. **(A)** BODIPY staining was used to assess LD accumulation in PLIN3-knockdown Huh7 and PLC/PRF/5 cells with OA stimulation. **(B)** Measurement of TG content in PLIN3-knockdown Huh7 and PLC/PRF/5 cells with OA stimulation. dBSA served as the control solvent. **(C)** Cell proliferation was assessed via a CCK-8 assay to evaluate the effect of PLIN3 knockdown on Huh7 and PLC/PRF/5 cells with OA stimulation. **(D, E)** Transwell migration and wound healing assays were performed to assess the migration of PLIN3-knockdown Huh7 and PLC/PRF/5 cells with OA stimulation. **(F)** F-actin staining of PLIN3-knockdown Huh7 and PLC/PRF/5 cells was performed to examine cytoskeletal changes with OA stimulation. Statistical significance is indicated as ∗*P* < 0.05, ∗∗*P* < 0.01, ∗∗∗*P* < 0.001, and ∗∗∗∗*P* < 0.0001. Figure S5

Figure S6 Knockdown of the four hub lipid droplet-associated genes (LDAGs: SET, CKAP4, RAP1B, and PISD) inhibits hepatocellular carcinoma cell proliferation and migration. **(A)** Quantitative PCR analysis of SET, CKAP4, RAP1B, and PISD mRNA expression in Huh7 and PLC/PRF/5 cells following knockdown. **(B)** The effect of hub LDAGs knockdown on cell proliferation was evaluated using CCK-8 assays in Huh7 and PLC/PRF/5 cells. **(C)** Migration assays were performed to assess the migratory capacity of Huh7 and PLC/PRF/5 cells following knockdown of the four hub LDAGs. The data were presented as mean ± standard deviation. Statistical significance is indicated as ∗*P* < 0.05, ∗∗*P* < 0.01, ∗∗∗*P* < 0.001, and ∗∗∗∗*P* < 0.0001. Figure S6

Figure S7 PLIN3 overexpression increases proliferation and migration of hepatocellular carcinoma cells under OA-stimulated conditions. **(A)** BODIPY staining was used to assess LD accumulation in PLIN3-overexpressing MHCC97H and HCCLM3 cells with OA stimulation. **(B)** Measurement of TG content in PLIN3-overexpressing MHCC97H and HCCLM3 cells with OA stimulation. **(C)** Cell proliferation was assessed via a CCK-8 assay to evaluate the effect of PLIN3 overexpression on MHCC97H and HCCLM3 cell growth with OA stimulation. **(D, E)** Transwell migration and wound healing assays were performed to assess the migration of PLIN3-overexpressing MHCC97H and HCCLM3 cells with OA stimulation. **(F)** F-actin staining was used to examine cytoskeletal alterations in PLIN3-overexpressing MHCC97H and HCCLM3 cells with OA stimulation. Statistical significance is indicated as ∗*P* < 0.05, ∗∗*P* < 0.01, ∗∗∗*P* < 0.001, and ∗∗∗∗*P* < 0.0001. Figure S7
