# Supplementary material for: Lipid droplet-associated gene signatures classify metabolic subtypes and identify PLIN3 as a key driver in hepatocellular carcinoma
Source: Genes Dis. 2026 Feb 3;13(5):102067. doi: 10.1016/j.gendis.2026.102067 (PMC13276161; doi:10.1016/j.gendis.2026.102067)
Supplement: Multimedia component 1 [file mmc1.docx]

**Supplemental Figure legends**

**Figure S1 Functional and expression analyses of lipid droplet-associated genes (LDAGs) in** hepatocellular carcinoma **(HCC). (A)** Illustration of the 122 LD-associated proteins identified via the AmiGO2 database. Squares represent the functional categories to which these LD proteins belong. **(B)** GO and KEGG pathway analyses of LD-associated proteins. **(C–E)** Heatmap showing the expression profiles of LDAGs in HCC tissues and non-tumor tissues, based on data from the TCGA-LIHC, ICGC-LIRI-JP, and GSE14520 datasets, respectively.

**Figure S2 Subtype and clustering analyses of lipid droplet-associated genes (LDAGs) in** hepatocellular carcinoma **(HCC). (A)** Cumulative distribution function (CDF) plot of clustering results based on LDAG expression patterns. **(B)** Delta area of the CDF for clustering results based on LDAG expression patterns. **(C)** Principal component analysis (PCA) of LDAG expression in the HCC subgroups. **(D)** Association between the three identified HCC clusters based on LDAGs and various clinical features, including sex, age, BMI, TNM stage, histologic grade, inflammation, fibrosis, and survival status. **(E–G)** Heatmap of the consensus matrix based on the expression of 122 LDAGs in the ICGC-LIRI-JP, GSE14520, and GSE116174 cohorts. **(H–J)** PCA of LDAG expression patterns across the three HCC subgroups from the ICGC-LIRI-JP, GSE14520, and GSE116174 cohorts. **(K–M)** Heatmap showing the expression patterns of LDAGs across the three subgroups in the ICGC-LIRI-JP, GSE14520, and GSE116174 cohorts. **(N–P)** Kaplan‒Meier survival analysis of the three subgroups based on the gene expression data of 122 LDAGs in the ICGC-LIRI-JP, GSE14520, and GSE116174 datasets. C1, Cluster 1; C2, Cluster 2; C3, Cluster 3. Statistical significance is indicated as ^*^*P* < 0.05, ^**^*P* < 0.01, ^***^*P* < 0.001, and ^****^*P* < 0.0001.

**Figure S3 Gene mutations and enrichment of pathways across the three-lipid droplet-associated genes (LDAGs)-associated** hepatocellular carcinoma **(HCC) subtypes.** Gene mutation profiles of the three LDAG-associated HCC clusters. **(A–C)** Oncoplots showing the top 25 genes with somatic mutations in C1 (A), C2 (B), and C3 (C) subtypes. **(D–F)** Hyperactive and hypoactive processes of KEGG-enriched pathways in C1 (D), C2 (E), and C3 (F) subtypes. **(G–I)** Hyperactive and hypoactive processes of hallmark gene sets in C1 (G), C2 (H), and C3 (I) subtypes.

**Figure S4 Expression and prognostic relevance of hub lipid droplet-associated genes (LDAGs) in** hepatocellular carcinoma **(HCC). (A)** Expression levels of the four hub LDAGs (SET, CKAP4, RAP1B, and PISD) in HCC tissues (T) compared with non-tumor tissues (N), as analyzed in the TCGA-LIHC dataset. **(B)** Association between the expression of the four hub LDAGs (SET, CKAP4, RAP1B, and PISD) and the OS rate in HCC patients, based on data from the TCGA-LIHC cohort. **(C)** mRNA levels of four hub LDAGs (SET, CKAP4, RAP1B, and PISD) in HCC tissue samples (T) and paired non-tumor tissues (N) were detected by quantitative PCR. **(D)** Expression levels of PLIN3 in HCC tissues compared with nontumor tissues were analyzed in the GSE45436 and GSE112790 datasets. **(E)** Analysis of the association between PLIN3 expression and various clinical features, including sex, BMI, TNM stage, histologic grade, inflammation, metastasis, and survival status, in HCC patients from the TCGA-LIHC dataset. **(F)** A nomogram was constructed based on a multivariate Cox regression analysis of PLIN3 expression and clinical characteristics to predict overall survival in HCC patients in terms of 1-year, 3-year, and 5-year survival. Statistical significance is indicated as "ns" (no significant difference); ^*^*P* < 0.05, ^**^*P* < 0.01, ^***^*P* < 0.001, and ^****^*P* < 0.0001.

**Figure S5 PLIN3 knockdown suppresses proliferation and migration of** hepatocellular carcinoma **cells under oleic acid (OA)-stimulated conditions. (A)** BODIPY staining was used to assess LD accumulation in PLIN3-knockdown Huh7 and PLC/PRF/5 cells with OA stimulation. **(B)** Measurement of TG content in PLIN3-knockdown Huh7 and PLC/PRF/5 cells with OA stimulation. dBSA served as the control solvent. **(C)** Cell proliferation was assessed via a CCK-8 assay to evaluate the effect of PLIN3 knockdown on Huh7 and PLC/PRF/5 cells with OA stimulation. **(D, E)** Transwell migration and wound healing assays were performed to assess the migration of PLIN3-knockdown Huh7 and PLC/PRF/5 cells with OA stimulation. **(F)** F-actin staining of PLIN3-knockdown Huh7 and PLC/PRF/5 cells was performed to examine cytoskeletal changes with OA stimulation. Statistical significance is indicated as ^*^*P* < 0.05, ^**^*P* < 0.01, ^***^*P* < 0.001, and ^****^*P* < 0.0001.

**Figure S6 Knockdown of the four hub lipid droplet-associated genes (LDAGs: SET, CKAP4, RAP1B, and PISD) inhibits** hepatocellular carcinoma **cell proliferation and migration. (A)** Quantitative PCR analysis of SET, CKAP4, RAP1B, and PISD mRNA expression in Huh7 and PLC/PRF/5 cells following knockdown. **(B)** The effect of hub LDAGs knockdown on cell proliferation was evaluated using CCK-8 assays in Huh7 and PLC/PRF/5 cells. **(C)** Migration assays were performed to assess the migratory capacity of Huh7 and PLC/PRF/5 cells following knockdown of the four hub LDAGs. The data were presented as mean ± standard deviation. Statistical significance is indicated as ^*^*P* < 0.05, ^**^*P* < 0.01, ^***^*P* < 0.001, and ^****^*P* < 0.0001.

**Figure S7 PLIN3 overexpression increases proliferation and migration of** hepatocellular carcinoma **cells under OA-stimulated conditions. (A)** BODIPY staining was used to assess LD accumulation in PLIN3-overexpressing MHCC97H and HCCLM3 cells with OA stimulation. **(B)** Measurement of TG content in PLIN3-overexpressing MHCC97H and HCCLM3 cells with OA stimulation. **(C)** Cell proliferation was assessed via a CCK-8 assay to evaluate the effect of PLIN3 overexpression on MHCC97H and HCCLM3 cell growth with OA stimulation. **(D, E)** Transwell migration and wound healing assays were performed to assess the migration of PLIN3-overexpressing MHCC97H and HCCLM3 cells with OA stimulation. **(F)** F-actin staining was used to examine cytoskeletal alterations in PLIN3-overexpressing MHCC97H and HCCLM3 cells with OA stimulation. Statistical significance is indicated as ^*^*P* < 0.05, ^**^*P* < 0.01, ^***^*P* < 0.001, and ^****^*P* < 0.0001.

**Figure S8** Differentially expressed genes **(DEGs) and functional enrichment analysis between** the low and high PLIN3 expression groups of hepatocellular carcinoma (HCC) patients from the TCGA-LIHC dataset. **(A) Identification of DEGs** between the low and high PLIN3 expression groups of HCC patients from the TCGA-LIHC dataset**. (B, C) Functional enrichment analyses of upregulated and downregulated DEGs, respectively. (D)** Correlation analysis between PLIN3 expression and key signaling pathway scores. Statistical significance is indicated as "ns" (no significant difference); ^*^*P* < 0.05, ^**^*P* < 0.01, ^***^*P* < 0.001, and ^****^*P* < 0.0001.

**Table S1** A detailed list of the 122 **lipid droplet**-associated proteins.

**Table S2** Clustering of **lipid droplet**-associated proteins based on their biological roles.

**Table S3** Detailed information of the datasets used in this study.

**Table S4** The expression profiles of the 122 **lipid droplet-associated genes (**LDAGs) across several hepatocellular carcinoma datasets.

**Table S5** Classification information of different clustering methods in TCGA-LIHC.

**Table S6** Cluster-specific differential expression genes of the associated hepatocellular carcinoma (HCC)**-lipid droplet (**LD) clusters.

**Table S7** Pathway differential analysis of GSVA in KEGG and Hallmark.

**Table S8** Cluster 1-specific drugs.

**Table S9** 67 potential drug targets in different subtypes and non-tumor tissues.

**Table S10** Correlation analysis between **lipid droplet-associated genes (**LDAGs) and non-LD differentially expressed genes (DEGs).

**Table S11** Pathway enrichment analysis of non-LD differentially expressed genes (DEGs) highly correlated with hub **lipid droplet-associated genes (**LDAGs).

**Table S12 RNA-sequencing data of between shPLIN3 and non-targeted control in Huh7 cells.**

**Table S13 KEGG Functional enrichment analysis of differentially expressed genes (DEGs) between shPLIN3 and non-targeted control in Huh7 cells.**

**Table S14 1028** differentially expressed genes **(DEGs) between PLIN3-low and PLIN3-high expression groups in HCC patients from the TCGA-LIHC cohort.**

**Table S15 KEGG Functional enrichment analysis of** differentially expressed genes **(DEGs) between PLIN3-low and PLIN3-high expression groups in** hepatocellular carcinoma **(HCC) patients from the TCGA-LIHC cohort.**
