# Supplementary figures and images for: Lipid droplet-associated gene signatures classify metabolic subtypes and identify PLIN3 as a key driver in hepatocellular carcinoma
Source: Genes Dis. 2026 Feb 3;13(5):102067. doi: 10.1016/j.gendis.2026.102067 (PMC13276161; doi:10.1016/j.gendis.2026.102067)

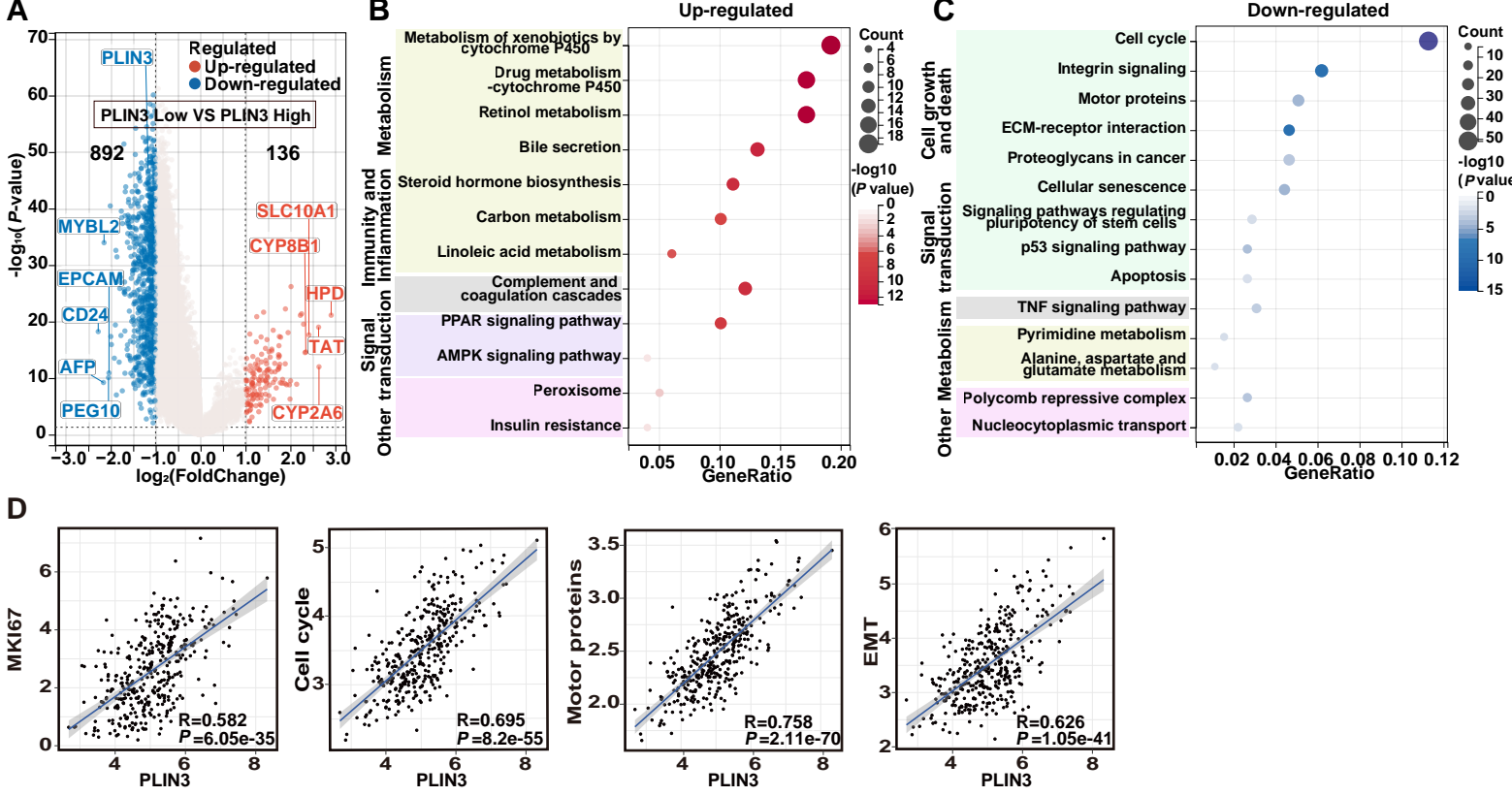

Supplement: Figure S8 — Differentially expressed genes (DEGs) and functional enrichment analysis between the low and high PLIN3 expression groups of hepatocellular carcinoma (HCC) patients from the TCGA-LIHC dataset. (A) Identification of DEGs between the low and high PLIN3 expression groups of HCC patients from the TCGA-LIHC dataset. (B, C) Functional enrichment analyses of upregulated and downregulated DEGs, respectively. (D) Correlation analysis between PLIN3 expression and key signaling pathway scores. Statistical significance is indicated as “ns” (no significant difference); ∗P < 0.05, ∗∗P < 0.01, ∗∗∗P < 0.001, and ∗∗∗∗P < 0.0001. [file mmc10.pdf]

A

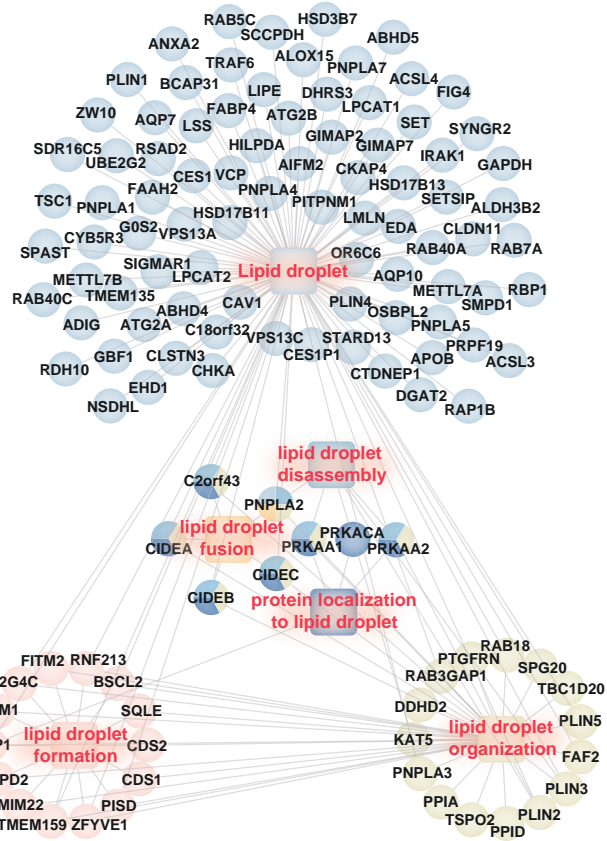

B

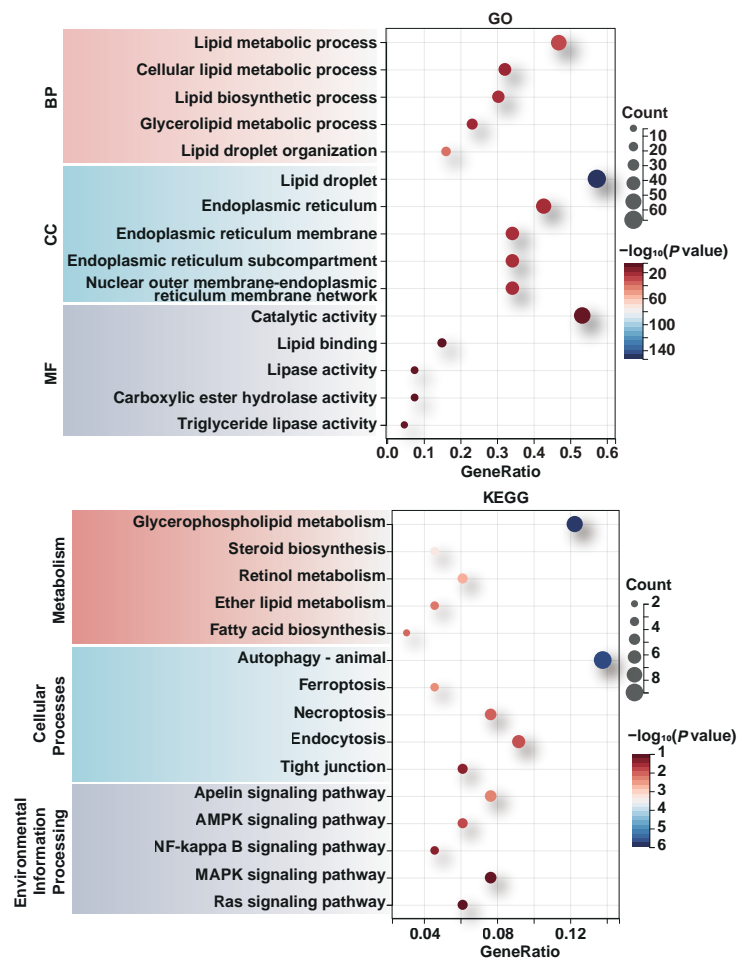

C

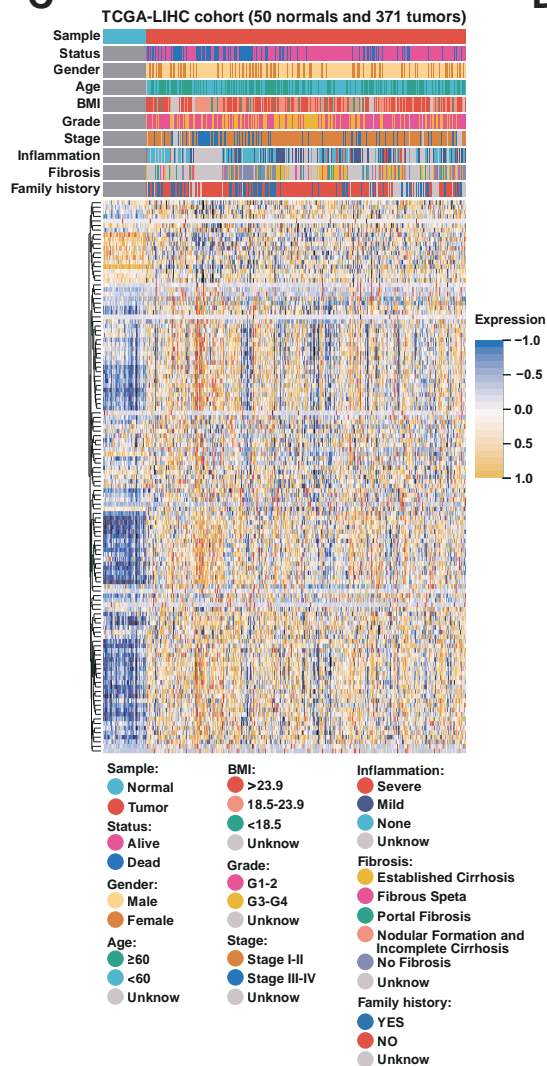

D

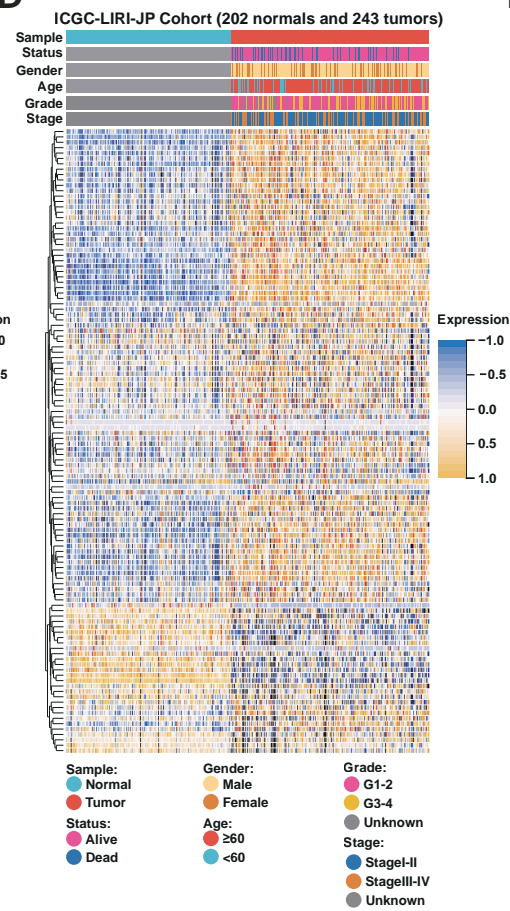

E

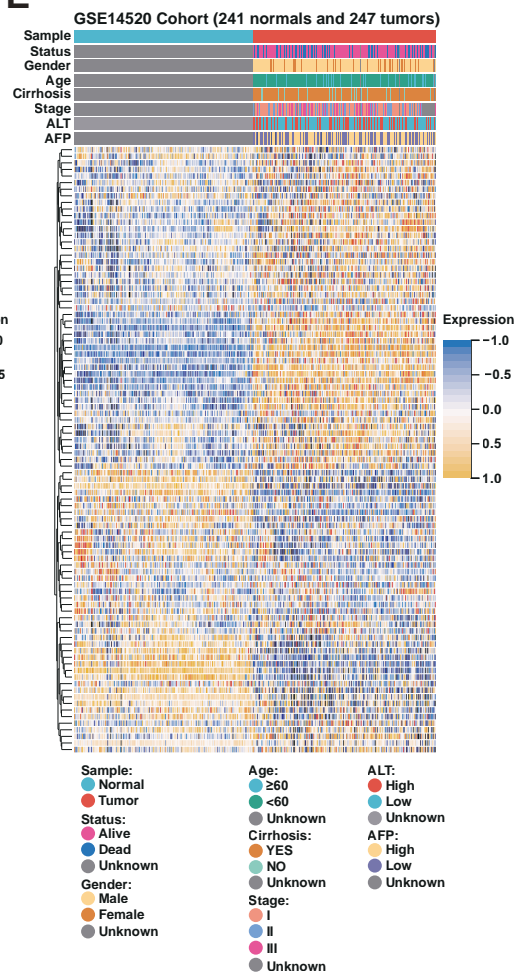

Supplement: Figure S1 — Functional and expression analyses of lipid droplet-associated genes (LDAGs) in hepatocellular carcinoma (HCC). (A) Illustration of the 122 LD-associated proteins identified via the AmiGO2 database. Squares represent the functional categories to which these LD proteins belong. (B) GO and KEGG pathway analyses of LD-associated proteins. (C–E) Heatmap showing the expression profiles of LDAGs in HCC tissues and non-tumor tissues, based on data from the TCGA-LIHC, ICGC-LIRI-JP, and GSE14520 datasets, respectively. [file mmc3.pdf]

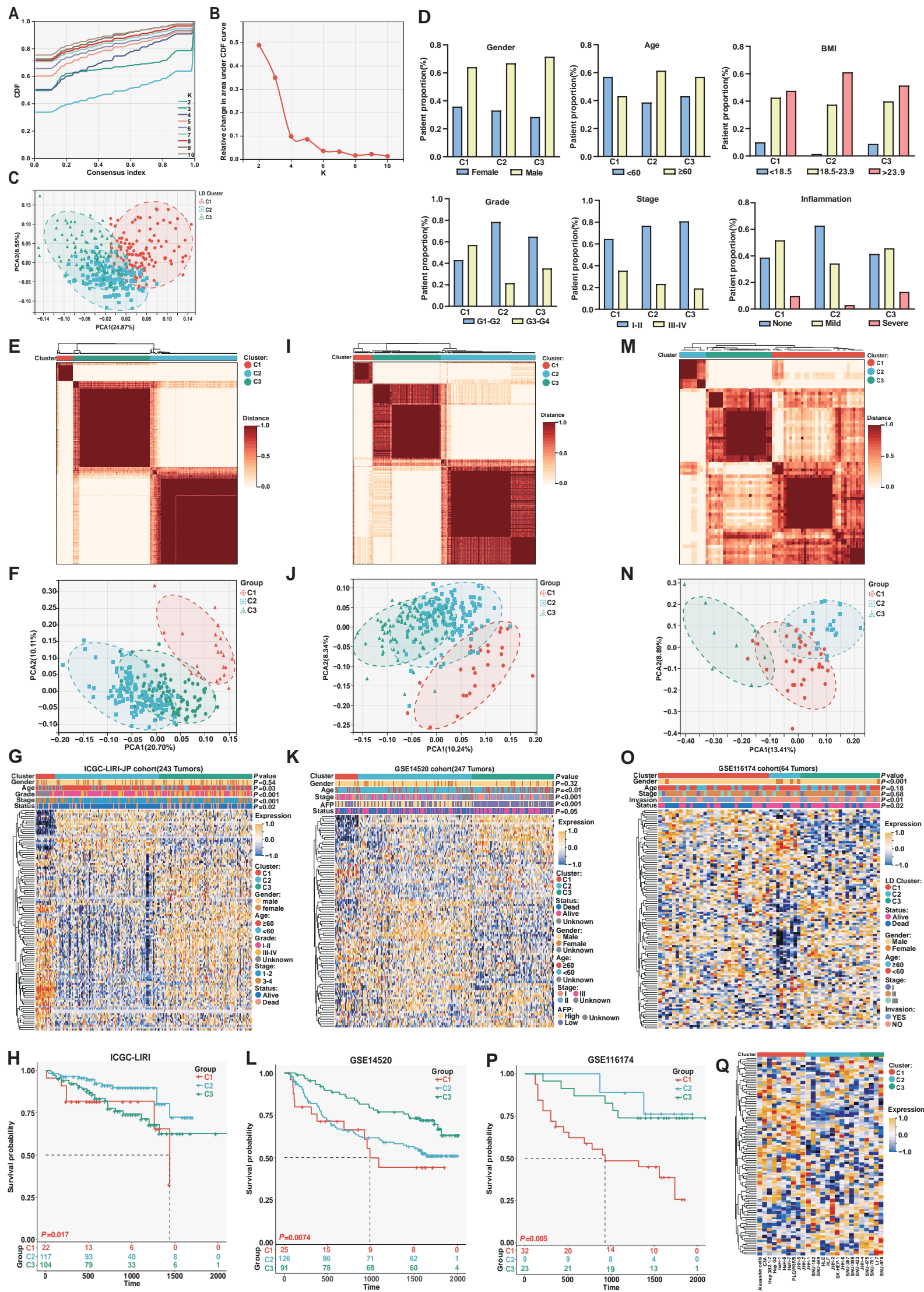

Supplement: Figure S2 — Subtype and clustering analyses of lipid droplet-associated genes (LDAGs) in hepatocellular carcinoma (HCC). (A) Cumulative distribution function (CDF) plot of clustering results based on LDAG expression patterns. (B) Delta area of the CDF for clustering results based on LDAG expression patterns. (C) Principal component analysis (PCA) of LDAG expression in the HCC subgroups. (D) Association between the three identified HCC clusters based on LDAGs and various clinical features, including sex, age, BMI, TNM stage, histologic grade, inflammation, fibrosis, and survival status. (E–G) Heatmap of the consensus matrix based on the expression of 122 LDAGs in the ICGC-LIRI-JP, GSE14520, and GSE116174 cohorts. (H–J) PCA of LDAG expression patterns across the three HCC subgroups from the ICGC-LIRI-JP, GSE14520, and GSE116174 cohorts. (K–M) Heatmap showing the expression patterns of LDAGs across the three subgroups in the ICGC-LIRI-JP, GSE14520, and GSE116174 cohorts. (N–P) Kaplan–Meier survival analysis of the three subgroups based on the gene expression data of 122 LDAGs in the ICGC-LIRI-JP, GSE14520, and GSE116174 datasets. C1, Cluster 1; C2, Cluster 2; C3, Cluster 3. Statistical significance is indicated as ∗P < 0.05, ∗∗P < 0.01, ∗∗∗P < 0.001, and ∗∗∗∗P < 0.0001. [file mmc4.pdf]

**A**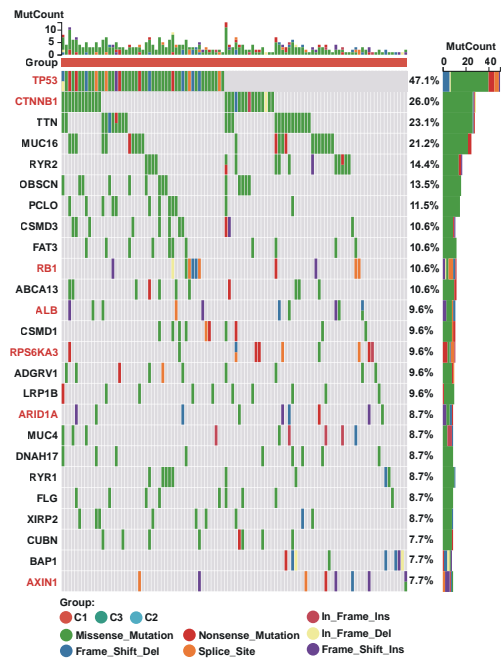**B**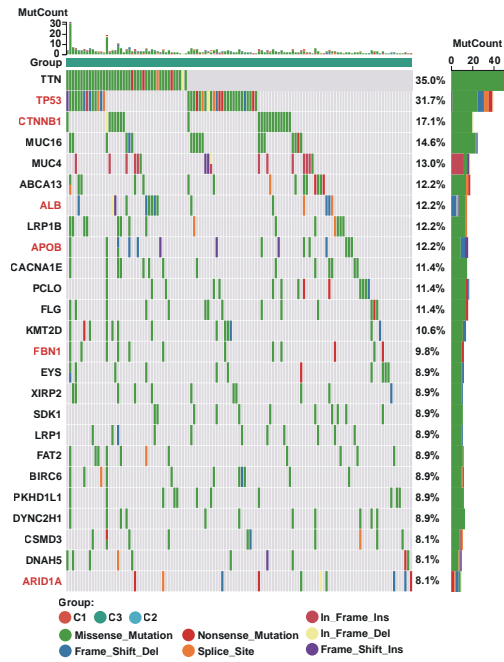**C**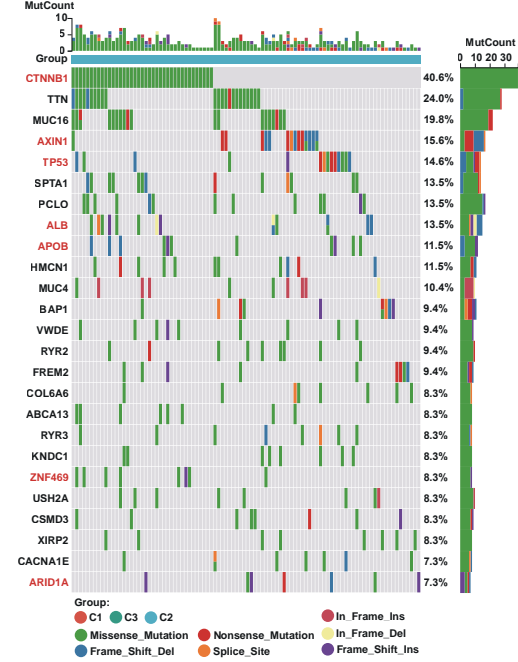**D**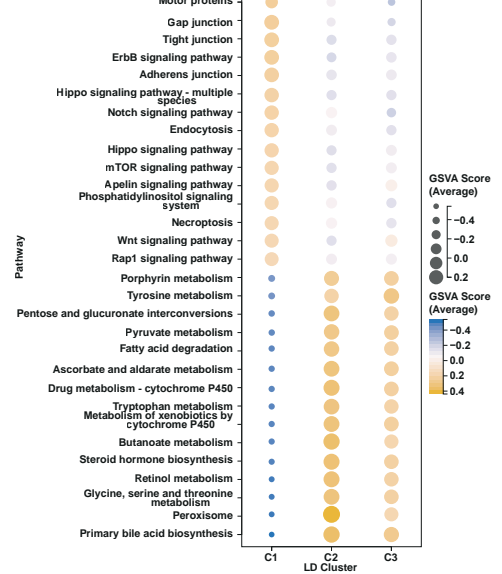**E**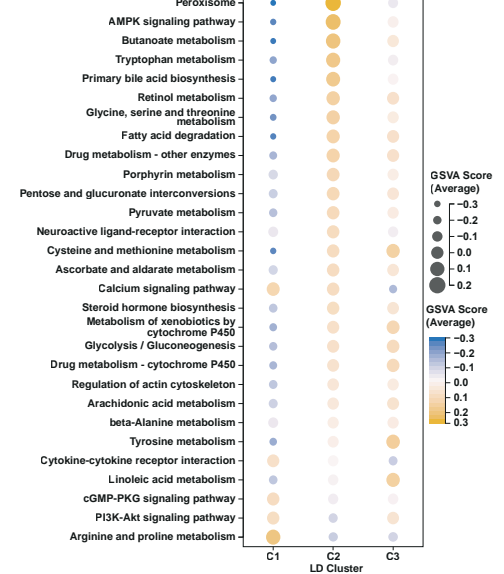**F**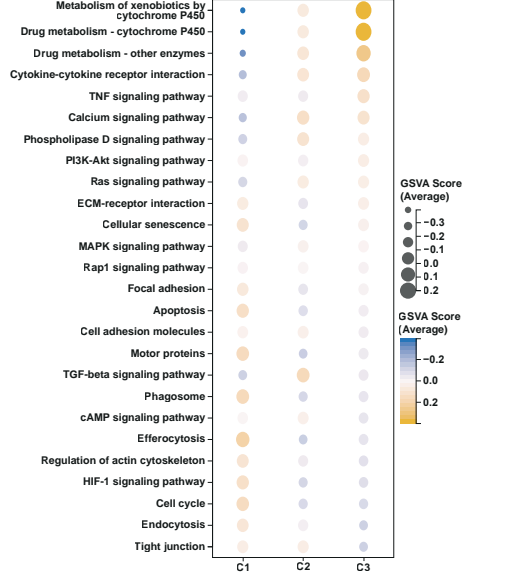**G**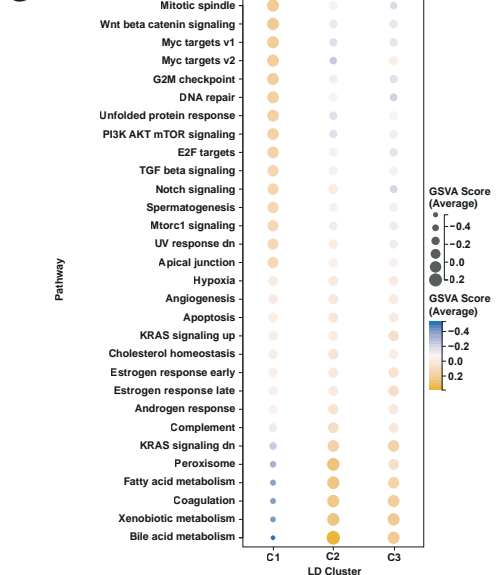**H**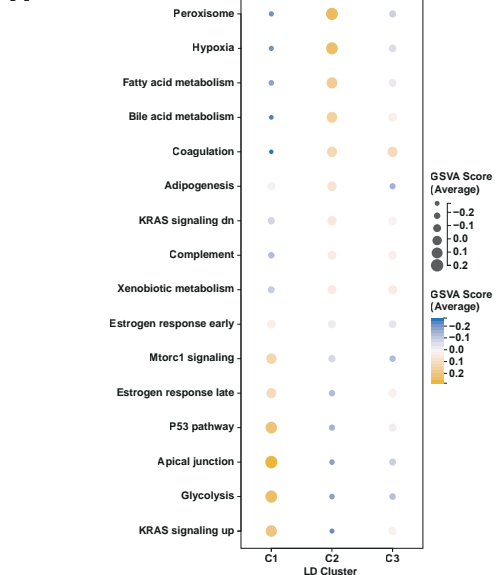**I**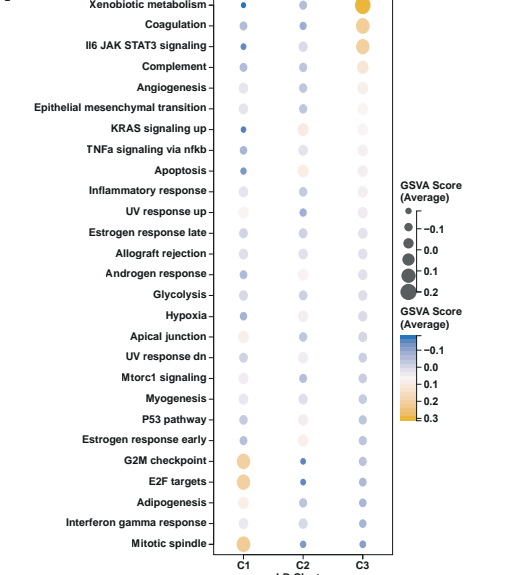

Supplement: Figure S3 — Gene mutations and enrichment of pathways across the three-lipid droplet-associated genes (LDAGs)-associated hepatocellular carcinoma (HCC) subtypes. Gene mutation profiles of the three LDAG-associated HCC clusters. (A–C) Oncoplots showing the top 25 genes with somatic mutations in C1 (A), C2 (B), and C3 (C) subtypes. (D–F) Hyperactive and hypoactive processes of KEGG-enriched pathways in C1 (D), C2 (E), and C3 (F) subtypes. (G–I) Hyperactive and hypoactive processes of hallmark gene sets in C1 (G), C2 (H), and C3 (I) subtypes. [file mmc5.pdf]

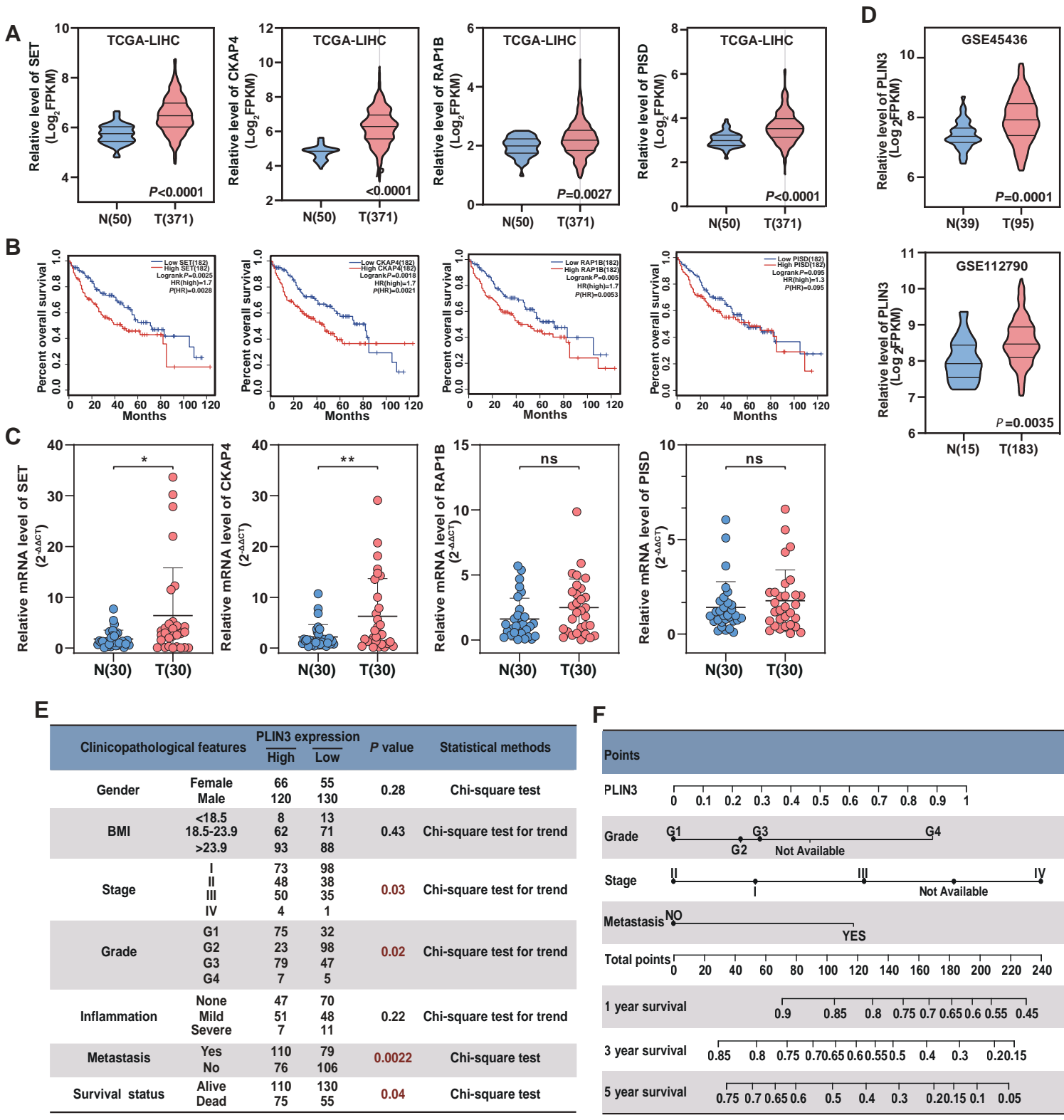

Supplement: Figure S4 — Expression and prognostic relevance of hub lipid droplet-associated genes (LDAGs) in hepatocellular carcinoma (HCC). (A) Expression levels of the four hub LDAGs (SET, CKAP4, RAP1B, and PISD) in HCC tissues (T) compared with non-tumor tissues (N), as analyzed in the TCGA-LIHC dataset. (B) Association between the expression of the four hub LDAGs (SET, CKAP4, RAP1B, and PISD) and the OS rate in HCC patients, based on data from the TCGA-LIHC cohort. (C) mRNA levels of four hub LDAGs (SET, CKAP4, RAP1B, and PISD) in HCC tissue samples (T) and paired non-tumor tissues (N) were detected by quantitative PCR. (D) Expression levels of PLIN3 in HCC tissues compared with nontumor tissues were analyzed in the GSE45436 and GSE112790 datasets. (E) Analysis of the association between PLIN3 expression and various clinical features, including sex, BMI, TNM stage, histologic grade, inflammation, metastasis, and survival status, in HCC patients from the TCGA-LIHC dataset. (F) A nomogram was constructed based on a multivariate Cox regression analysis of PLIN3 expression and clinical characteristics to predict overall survival in HCC patients in terms of 1-year, 3-year, and 5-year survival. Statistical significance is indicated as “ns” (no significant difference); ∗P < 0.05, ∗∗P < 0.01, ∗∗∗P < 0.001, and ∗∗∗∗P < 0.0001. [file mmc6.pdf]

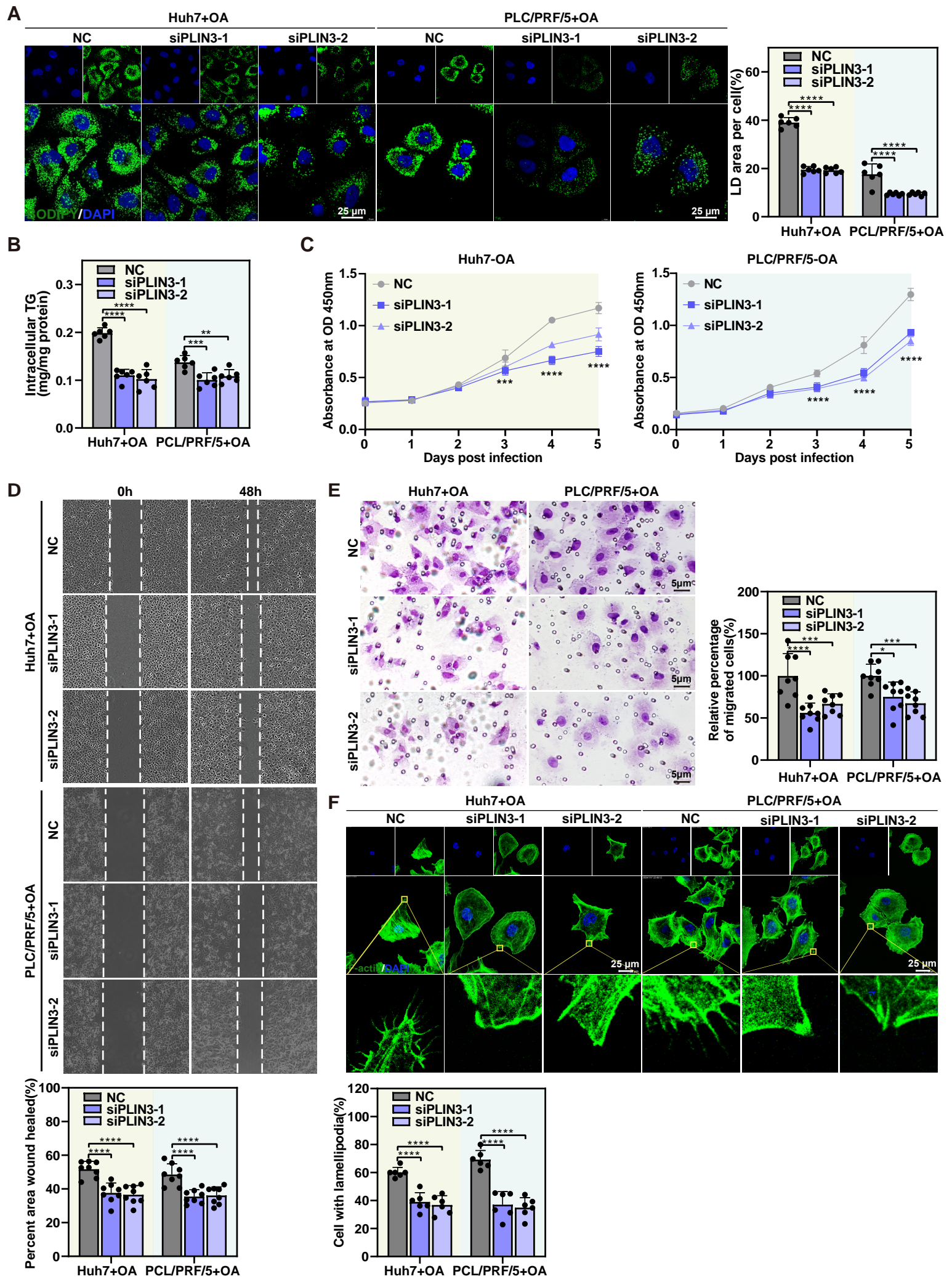

Supplement: Figure S5 — PLIN3 knockdown suppresses proliferation and migration of hepatocellular carcinoma cells under oleic acid (OA)-stimulated conditions. (A) BODIPY staining was used to assess LD accumulation in PLIN3-knockdown Huh7 and PLC/PRF/5 cells with OA stimulation. (B) Measurement of TG content in PLIN3-knockdown Huh7 and PLC/PRF/5 cells with OA stimulation. dBSA served as the control solvent. (C) Cell proliferation was assessed via a CCK-8 assay to evaluate the effect of PLIN3 knockdown on Huh7 and PLC/PRF/5 cells with OA stimulation. (D, E) Transwell migration and wound healing assays were performed to assess the migration of PLIN3-knockdown Huh7 and PLC/PRF/5 cells with OA stimulation. (F) F-actin staining of PLIN3-knockdown Huh7 and PLC/PRF/5 cells was performed to examine cytoskeletal changes with OA stimulation. Statistical significance is indicated as ∗P < 0.05, ∗∗P < 0.01, ∗∗∗P < 0.001, and ∗∗∗∗P < 0.0001. [file mmc7.pdf]

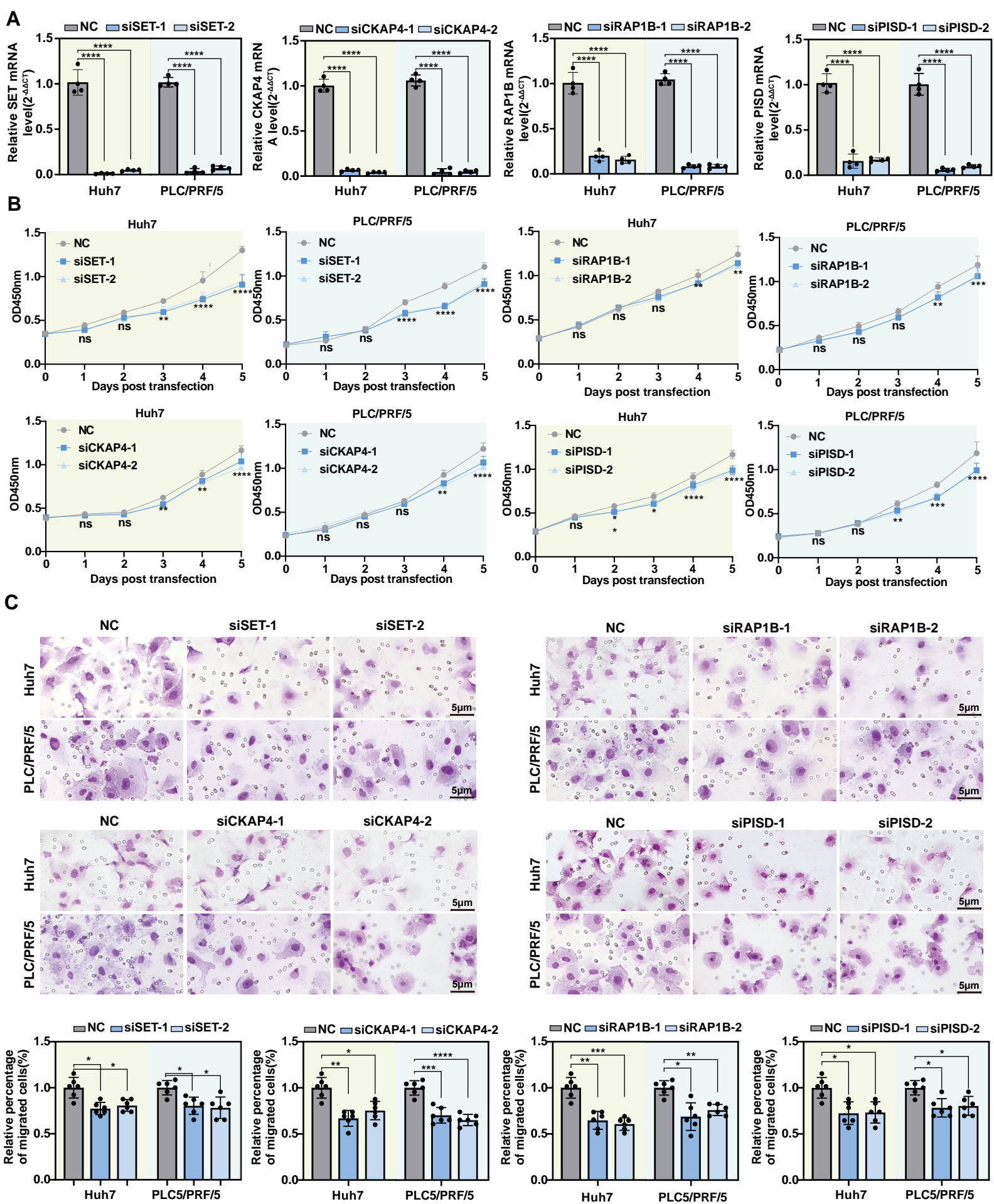

Supplement: Figure S6 — Knockdown of the four hub lipid droplet-associated genes (LDAGs: SET, CKAP4, RAP1B, and PISD) inhibits hepatocellular carcinoma cell proliferation and migration. (A) Quantitative PCR analysis of SET, CKAP4, RAP1B, and PISD mRNA expression in Huh7 and PLC/PRF/5 cells following knockdown. (B) The effect of hub LDAGs knockdown on cell proliferation was evaluated using CCK-8 assays in Huh7 and PLC/PRF/5 cells. (C) Migration assays were performed to assess the migratory capacity of Huh7 and PLC/PRF/5 cells following knockdown of the four hub LDAGs. The data were presented as mean ± standard deviation. Statistical significance is indicated as ∗P < 0.05, ∗∗P < 0.01, ∗∗∗P < 0.001, and ∗∗∗∗P < 0.0001. [file mmc8.pdf]

**A**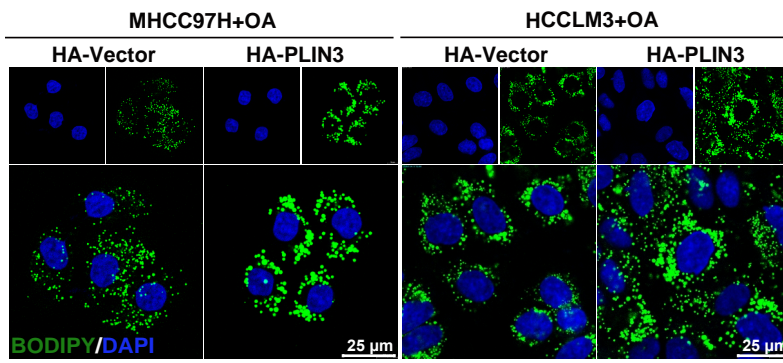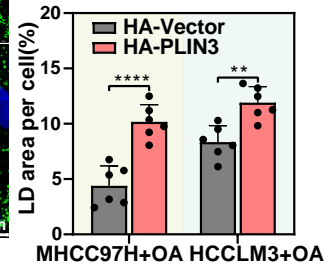**B**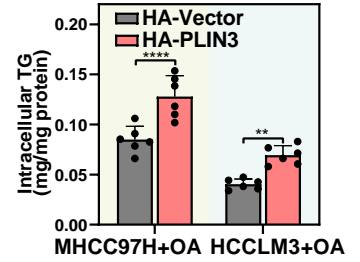**C**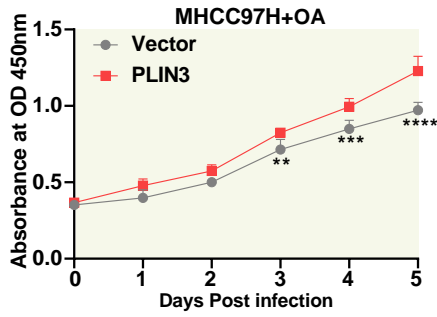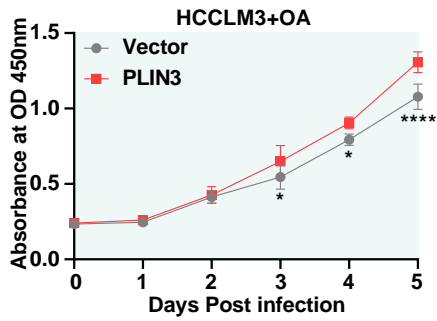**D**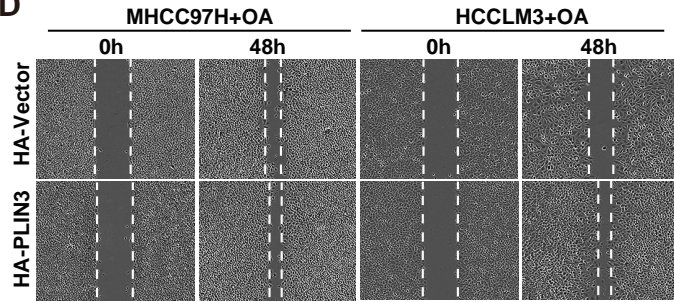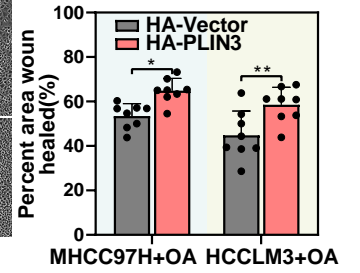**E**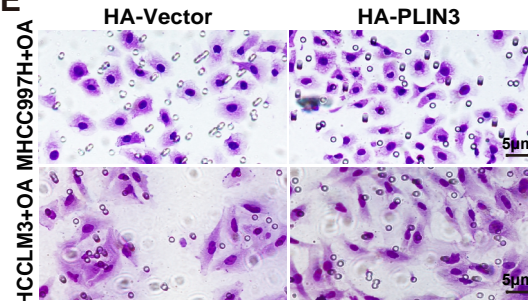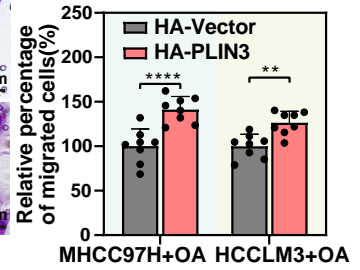**F**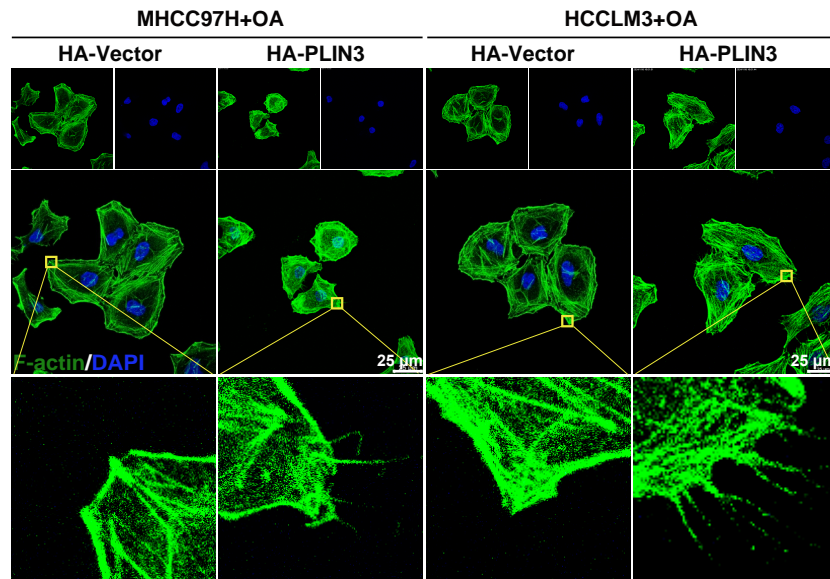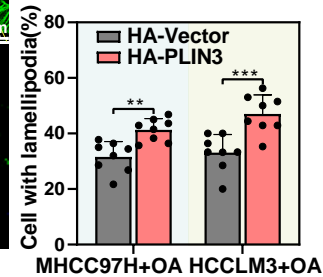

Supplement: Figure S7 — PLIN3 overexpression increases proliferation and migration of hepatocellular carcinoma cells under OA-stimulated conditions. (A) BODIPY staining was used to assess LD accumulation in PLIN3-overexpressing MHCC97H and HCCLM3 cells with OA stimulation. (B) Measurement of TG content in PLIN3-overexpressing MHCC97H and HCCLM3 cells with OA stimulation. (C) Cell proliferation was assessed via a CCK-8 assay to evaluate the effect of PLIN3 overexpression on MHCC97H and HCCLM3 cell growth with OA stimulation. (D, E) Transwell migration and wound healing assays were performed to assess the migration of PLIN3-overexpressing MHCC97H and HCCLM3 cells with OA stimulation. (F) F-actin staining was used to examine cytoskeletal alterations in PLIN3-overexpressing MHCC97H and HCCLM3 cells with OA stimulation. Statistical significance is indicated as ∗P < 0.05, ∗∗P < 0.01, ∗∗∗P < 0.001, and ∗∗∗∗P < 0.0001. [file mmc9.pdf]
